# Thiosemicarbazone-Based Compounds: Cancer Cell Inhibitors with Antioxidant Properties

**DOI:** 10.3390/molecules30092077

**Published:** 2025-05-07

**Authors:** Olga Garbuz, Emil Ceban, Dorin Istrati, Nadejda Railean, Ion Toderas, Aurelian Gulea

**Affiliations:** 1 Laboratory of Systematics and Molecular Phylogenetics, Institute of Zoology, Moldova State University, 1 Academiei Street, MD-2028 Chisinau, Moldova; 2 Department of Urology and Surgical Nephrology, University of Medicine and Pharmacy “Nicolae Testemitanu”, 165 Stefan cel Mare si Sfant Bd., MD-2004 Chisinau, Moldova; 3 Laboratory of Advanced Materials in Biopharmaceutics and Technics, Institute of Chemistry, Moldova State University, 60 Mateevici Street, MD-2009 Chisinau, Moldova

**Keywords:** anticancer activity, antioxidant activities, selectivity, synthetic compound, thiosemicarbazone, coordination compounds

## Abstract

Thiosemicarbazone-based compounds have attracted significant attention in recent years due to their potential as inhibitors of cancer cell proliferation. They not only exhibit strong antiproliferative effects but also possess antioxidant properties that are crucial in combating oxidative stress linked to cancer progression. This review highlights specific compounds that not only exhibit significantly higher antiproliferative activities but also demonstrate lower toxicity compared to traditional chemotherapy agents. This is important because it suggests that these compounds could provide better treatment options while reducing the side effects often associated with chemotherapy. A detailed analysis of the structure–activity relationships (SARs) reveals that the unique structural features of these compounds play a crucial role in their enhanced effectiveness. Understanding which molecular characteristics contribute to improved activity will be key for future compound design. The findings from this study emphasize the need for further exploration and development of these novel agents. By investigating their biological mechanisms and optimizing their structures, researchers can improve cancer treatment strategies, providing safer and more effective options for patients. Despite substantial previous research on thiosemicarbazones and isothiosemicarbazones, the field still holds many unknowns and opportunities for discovery. Studying coordination chemistry with 3*d* metal ions and strategically modifying their inner structures may lead to new compounds with promising biological activities and selectivity. Overall, exploring thiosemicarbazones and isothiosemicarbazones as innovative pharmacological agents against cancer could unlock their full potential, significantly enhancing cancer treatment protocols and improving patient survival rates.

## 1. Introduction

Cancer remains one of the leading causes of death globally, with an alarming rise in incidence rates across various populations. The uncontrolled proliferation of cancer cells and their ability to metastasize pose significant challenges in effective treatment, highlighting the urgent need for novel therapeutic strategies [1]. Despite advancements in chemotherapy, traditional agents often exhibit severe side effects and limited selectivity, necessitating the exploration of new compounds [2] that can selectively target cancer cells while minimizing harm to healthy tissues [3].

Current research trends indicate a growing interest in the development of synthetic anticancer agents, particularly those that integrate metal coordination into their structure. Coordination compounds of transition metals have garnered attention due to their potential to enhance biological activity through unique interactions with cellular targets. Among these, thiosemicarbazones have shown promise as ligands, leading to the formation of complexes that may improve antiproliferative efficacy and selectivity against various cancer cell lines [4].

Controversy exists regarding the mechanisms through which different classes of anticancer agents exert their effects. While some studies emphasize the role of oxidative stress and apoptosis induction, others explore alternative pathways, such as DNA damage and cell cycle disruption. This divergence underscores the complexity of cancer biology and calls for comprehensive investigations to elucidate the precise mechanisms of action for new compounds [5,6].

In this review, we have comprehensively covered the evaluation of synthetic anticancer compounds, focusing on coordination complexes of 3*d* metals with thiosemicarbazone ligands. This study aimed to assess these compounds for their anticancer activity, selectivity, and antioxidant properties. Through detailed exploration, the research highlights the impact of metal coordination on enhancing biological activity. This review also discusses the induction of apoptosis in cancer cells as a mechanism of action and explores the relationship between antiproliferative and antioxidant activities [7], indicating that compounds with robust antioxidant properties may also exhibit significant anticancer effects [8].

## 2. Anticancer and Antioxidant Properties of the Synthetic Compounds and the Influence of Structure on Their Activity

### 2.1. Antiproliferative and Antioxidant Activities in Drug Development

Antiproliferative activity (APA) and antioxidant activity (AOA) are two important parameters frequently used to evaluate the biological activity of compounds, particularly in the context of drug development [9].

Oxidative damage to DNA is considered a significant factor in carcinogenesis, aging, and neurological degeneration. Research has shown that such damage accumulates in cancerous tissues. For instance, higher levels of oxidative base damage have been observed in lung cancer tissues compared to surrounding normal tissues. Additionally, studies report a significant increase in oxidative DNA damage markers such as 8-oxoG, 8-hydroxyadenine, and 2,6-diamino-4-hydroxy-5-formamidopyrimidine in breast cancer tissues compared to normal tissues [10,11]. This damage accumulation correlates with an increased cancer risk with age, suggesting that cancer might be viewed as a degenerative disease linked to aging [12,13]. Evidence supports the accumulation of oxidative DNA damage with age, primarily through increased levels of 8-oxoG [14].

Antioxidant activity refers to the ability of compounds to neutralize free radicals and prevent oxidative damage to cells. By donating electrons or hydrogen atoms, antioxidants reduce the reactivity of free radicals, helping cells defend against oxidative stress. AOA is commonly evaluated using methods like ABTS^•+^ [15], DPPH^•^ [16], and ORAC [17], among others, with Trolox used as the standard for comparison (Table 1).

Anticancer activity is defined by the ability of compounds to inhibit the proliferative of cancer cells. Compounds exhibiting antiproliferative activity may induce cell cycle arrest, promote apoptosis, or alter cell-signaling pathways involved in growth regulation. Some antioxidants may indirectly influence APA by preventing DNA damage and reducing inflammatory processes that can contribute to tumor growth. APA is typically assessed using assays such as MTT and resazurin, among others, with Doxorubicin [18,19] and Cisplatin [20,21] used as standards for comparison (Table 1).

Research indicates that substances with high antioxidant activity may also possess anticancer properties. Various studies and reviews support this notion by examining the role of antioxidants in scavenging free radicals [22] and reducing oxidative stress, which is a contributor to the development of cancer. However, this is not always the case; the presence of antioxidant activity does not guarantee that a compound will exhibit significant antiproliferative activity. For instance, some compounds may effectively neutralize free radicals but have little to no direct impact on cells or show insufficient effectiveness at high concentrations [23,24].

Thus, comparing antioxidant and antiproliferative activities is essential for understanding the potential therapeutic effects of various compounds [25,26]. A deeper exploration of the relationship between AOA and antiproliferative activity may lead to the development of more effective drugs that can simultaneously protect cells from oxidative stress and inhibit the growth of cancer cells [27,28]. The development of such compounds requires a thorough analysis of their properties and mechanisms of action, as well as experimental studies to confirm their activity and effectiveness [29].

High systemic toxicity and drug resistance remain a major challenge for modern medicine in the management of cancer despite the significant progress made in anticancer therapy. Chemotherapy can produce severe side effects caused by its cytotoxic effect on normal cells. This limits their use, and it is an indication to reduce the drug dose, or interrupt and even cease the treatment. Therefore, it is important that anticancer drugs exert antiproliferative and cytotoxic activity in tumor cells without affecting normal tissues, so the principal need in the chemoprevention of cancer remains the discovery of new agents that are effective and safe [30].

Doxorubicin is recognized as one of the most potent FDA-approved chemotherapeutic agents and is a frontline drug. However, it is known for causing toxicity to most major organs, particularly cardiotoxicity, as it induces toxic damage to the mitochondria of cardiomyocytes, leading to increased oxidative stress [18,19].

Platinum-based anticancer drugs also play a leading role in the treatment of various malignant tumors, but severe side effects such as nephrotoxicity, neurotoxicity, and drug resistance have limited their wide range of clinical applications [31,32]. This has stimulated extensive research and has promoted chemists to establish alternative approaches on the basis of using endogenous metals to improve the pharmacological properties. Among the many bio-essential metals, copper coordination compounds are regarded as promising alternatives to platinum coordination compounds as anticancer drugs because copper is biocompatible and exhibits many significant roles in biological systems [33]. Also, copper shows altered metabolism in cancer cells and a differential response between normal and tumor cells [34,35]. It is proven that the concentration of copper in cancerous tissues exceeds that of normal tissue, and the sequestration of copper can prevent the establishment of new blood vessels [36]. Therefore, cancer cells may represent a suitable, selective target for copper-based agents [37,38].

In recent years, a large number of synthetic copper(II) coordination compounds of thiosemicarbazone ligands have been reported to act as pharmacological agents and as potential anticancer and cancer-inhibiting agents, and they have been found to be active both *in vitro* and *in vivo* [39].

Thiosemicarbazone is a class of organic compounds that possesses a wide spectrum of biological activities and medical properties [40]. Thiosemicarbazones contain a wide range of donor atoms and, therefore, can form coordination compounds with transition metal ions [41].

Different models, such as cancer cell lines, explants of tumor and normal tissues, and enzyme systems, are used to develop and test anticancer substances, but the selection of antitumor substances is carried out mainly on various cancer cell lines [42,43].

The investigation of anticancer activity in compounds is a critical step in the search for effective drug therapies to combat cancer. As cancer remains one of the leading causes of mortality worldwide, the development of new treatments is essential. Assessing the antiproliferative activity of compounds can serve as a foundational approach for the initial screening of substances that may eventually become candidates for drug development [44,45,46,47,48].

Research has demonstrated the antiproliferative and antioxidant properties of synthetic compounds, specifically thiosemicarbazones, and their 3*d* metal coordination complexes involving Cr(III), Mn(II), Fe(III), Co(III), Ni(II), Cu(II), and Zn(II) synthesized in research by acad. A. Gulea et al. [48] from the Laboratory of Advanced Materials in Biopharmaceutics and Technics of the Moldova State University, Institute of Chemistry. These findings offer valuable insights for preliminary screening of these substances for anticancer activity, potentially paving the way for the development of new therapeutic agents in oncology.

Many antitumor drugs work by inducing DNA damage, particularly effective during the S phase of the cell cycle when DNA replication occurs. To study this effect, cells in the exponential growth phase are commonly used. The IC_50_ value, or half maximal inhibitory concentration, serves as a critical metric for evaluating the efficacy of compounds in inhibiting cell proliferation. The selectivity index was determined using normal cell lines, specifically MDCK, to assess the compounds’ selectivity towards cancerous versus non-cancerous cells. This measurement is essential for determining the optimal therapeutic dose, providing a comparative assessment framework for the tested substances [45].

The results presented in Table 1 reveal that doxorubicin and cisplatin exhibit high toxicity, indicated by a low selectivity index ranging from 0.05 to 1.8. Both anticancer agents show significant efficacy against the studied several cancer cell lines HeLa, RD, and BxPC-3, with doxorubicin demonstrating superior activity compared to cisplatin. In addition, both compounds possess antioxidant properties, with doxorubicin surpassing the activity of Trolox. A correlation exists between the antioxidant and anticancer activities of doxorubicin and cisplatin, particularly in regard to their effects on the HeLa and BxPC-3 cancer cell lines [45].

**Table 1:** Antiproliferative activity of the standard drugs against HeLa, RD, and BxPC-3 cancer cell lines, as well as MDCK cell lines. ABTS^•+^ scavenging activity of DOXO, CDDP, and Trolox.

Compound	ABTS IC_50_ (µM)	SI	BxPC-3 IC_50_ (µM)	SI	RD IC_50_ (µM)	SI	HeLa IC_50_ (µM)	MDCK IC_50_ (µM)	Reference
**DOXO**	12.0	1.8	6.0	0.7	16.2	1.8	6.2	10.8	[47,48]
**CDDP**	43.0	0.1	11.2	0.3	4.6	0.05	30.9	1.5	[47,48]
**Trolox**	33.3	-	-	-	-	-	-	-	[47,48]

Average results of three experiments, SEM < ±3%.

### 2.2. Antiproliferative and Antioxidant Activities of Thiosemicarbazones

Thiosemicarbazones are a class of organic compounds formed by the reaction of thiosemicarbazide with aldehydes or ketones. Their general structure is represented by the formula R_1_R_2_C = NNH-C(S)NH_2_, where R_1_ and R_2_ are organic substituents. These compounds are known for their ability to form chelates by creating stable coordination compounds with metal ions, which significantly enhances their chemical reactivity [46].

The ability of thiosemicarbazones to bind with metal ions increases their chemical activity and facilitates the formation of stable coordination compounds, often exhibiting high biological activity enhanced by interactions with metals. Thiosemicarbazones can exist in various tautomeric forms, allowing them to adapt their structure for optimal interaction with biological targets and free radicals. This structural flexibility expands their ability to participate in various chemical reactions [47].

Thiosemicarbazones can inhibit the proliferation of cancer cells by inducing apoptosis or cell cycle arrest. Thiosemicarbazones can effectively neutralize free radicals due to their structure containing nitrogen and sulfur. These characteristics make thiosemicarbazones promising for further research. The results regarding the antioxidant and antiproliferative activities of the thiosemicarbazones, expressed in IC_50_ values, are presented in Table 2 [48].

The structural diversity of thiosemicarbazones plays a pivotal role in determining their anticancer and antioxidant activities. Variations in substituents and the arrangement of functional groups significantly influence the biological efficacy of these compounds.

The most pronounced antiproliferative activity against cancer cells is observed in the following thiosemicarbazone ligands: *N*-(3-methoxyphenyl)-2-[1-(pyridin-2-yl)ethylidene]hydrazinecarbothioamide, with IC_50_ ≤ 0.1 µM for BxPC-3, IC_50_ = 11.6 µM for RD, and IC_50_ = 5.8 µM for HeLa; *N*-(4-methoxyphenyl)-2-[1-(pyridin-2-yl)ethylidene]hydrazinecarbothioamide, with IC_50_ ≤ 0.1 µM for BxPC-3, IC_50_ = 11.2 µM for RD, and IC_50_ = 12.3 µM for HeLa; *N*-cyclohexyl-2-[1-(pyridin-3-yl)ethylidene]hydrazinecarbothioamide, with IC_50_ = 0.6 µM for BxPC-3 and IC_50_ = 10.3 µM for HeLa; *N*-(2-methoxyphenyl)-2-(pyridin-2-ylmethylidene)hydrazinecarbothioamide, with IC_50_ = 0.2 µM for BxPC-3 and IC_50_ = 23 µM for RD [49] (Table 2).

The antiproliferative activity of these thiosemicarbazone ligands can be attributed to several properties’ structural features. For instance, the cyclohexyl group in *N*-cyclohexyl-2-[1-(pyridin-3-yl)ethylidene]hydrazinecarbothioamide increases lipophilicity, facilitating cellular membrane penetration and allowing for more effective targeting of intracellular targets [50]. The pyridine ring provides additional π-π interactions with biological targets such as proteins [48].

Methoxyphenyl groups in ligands like *N*-(2-methoxyphenyl) and *N*-(3-methoxyphenyl) increase electron donation and bond polarizability, easing cellular interactions. Ligands containing alkenyl groups, such as *N*-(prop-2-en-1-yl), enhance reactivity and increase the chances of specific binding, as unsaturated fragments can participate in reactions via double bonds [51]. Hydroxyl groups in ligands like 2-[1-(2-hydroxyphenyl)ethylidene] facilitate the formation of hydrogen bonds and improve interactions with proteins that influence cell adhesion and division [52]. The introduction of *methoxy*- or *dimethyl*- groups at the *meta* and *para* positions reduces steric hindrance and results in optimal binding effects that influence interactions in cancer cell signaling pathways [53].

Molecular geometry, defined by the spatial arrangement of functional groups, ensures optimal interactions with cellular components such as DNA and enzymes involved in cell division [54]. Thus, structural features, including the diversity and positioning of functional groups, as well as spatial geometry, play a crucial role in providing high antiproliferative activity in thiosemicarbazides [55]. These properties enhance their potential for the specific targeting of cancer cells with minimal impact on normal cells [48].

All thiosemicarbazones in Table 2 exhibit high antioxidant activity, showing ABTS^•+^ scavenging activity in the concentration (IC_50_) range of 5 to 22 µM, surpassing that of Trolox, attributed to their distinct structural features and chemical properties. The impact of these structural characteristics on the antioxidant activity of thiosemicarbazones includes the presence of hydroxyl groups within phenolic structures, which allows these compounds to effectively donate hydrogen atoms and neutralize free radicals. This is because phenolic hydroxyl groups have a high capacity to stabilize charge by delocalizing electron density across the aromatic ring [56,57].

The placement of hydroxyl groups in the para position enhances stabilization through resonance and facilitates specific charge distribution, thus improving the ability of compounds to neutralize radicals. Methyl groups can increase the electron density on the ring, thereby improving the compound’s interaction with radicals. They also provide a hydrophobic effect, which can influence solubility and, consequently, the bioavailability of these compounds [58].

The presence of sulfur and nitrogen in the thiocarbamide group further enhances antioxidant activity, as these elements can participate in charge delocalization and radical stabilization. Together, these structural elements contribute to the high antioxidant activity of thiosemicarbazones [59]. Their ability to effectively neutralize free radicals and protect cells from oxidative stress makes them more active compared to Trolox [47]. Thus, the combination of hydroxyl and methyl groups in their specific arrangement leads to enhanced antioxidant properties, providing high efficacy in biological systems [57,60,61,62,63,64,65,66,67,68,69,70,71].

The most pronounced antioxidant activity [47,72,73,74,75,76,77,78,79,80,81,82] is observed in the following thiosemicarbazones: *N*-cyclohexyl-2-[1-(pyridin-3-yl)ethylidene]hydrazinecarbothioamide, with IC_50_ = 5 µM; *N*-cyclohexyl-2-(2-hydroxybenzylidene)hydrazinecarbothioamide, with IC_50_ = 6.7 µM; 2-(2,3-dihydroxybenzylidene)-*N*-(prop-2-en-1-yl)hydrazinecarbothioamide, with IC_50_ = 5.7 µM; 2-(2,4-dihydroxybenzylidene)-*N*-(prop-2-en-1-yl)hydrazinecarbothioamide, with IC_50_ = 5.5 µM; *N*-(3-methylphenyl)-2-[1-(pyridin-2-yl)ethylidene]hydrazinecarbothioamide, with IC_50_ = 5.1 µM; *N*-(2-methoxyphenyl)-2-(pyridin-2-ylmethylidene)hydrazinecarbothioamide, with IC_50_ = 6.3 µM; and *N*-[4-({[2-(2-hydroxybenzylidene)hydrazinyl]carbonothioyl}amino)phenyl]acetamide, with IC_50_ = 6.6 µM (Table 2) [48].

**Table 2:** Antiproliferative activity of thiosemicarbazones against BxPC-3, RD, and HeLa cancer cell lines, as well as the MDCK normal cell line. ABTS^•+^ scavenging activity of the compounds.

	Formula	ABTS IC_50_ (µM)	BxPC-3 IC_50_ (µM)	RD IC_50_ (µM)	HeLa IC_50_ (µM)	MDCK IC_50_ (µM)	Reference
**L^1^**	** 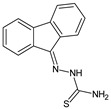 **	18.4	≥100	≥100	≥100	≥100	[47,48]
**L^2^**	** 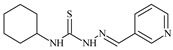 **	21.0	≥100	≥100	≥100	≥100	[47,48]
**L^3^**	** 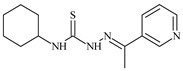 **	5.0 ± 0.2	0.60	47.7	10.3	35.8	[47,48]
**L^4^**	** 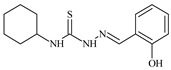 **	6.7	≥100	≥100	≥100	≥100	[47,48]
**L^5^**	** 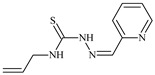 **	14.2	≥100	1.1	≥100	≥100	[46,47,48,83]
**L^6^**	** 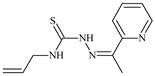 **	11.6	0.8	100.4	4.5	≥100	[46,47,48,83]
**L^7^**	** 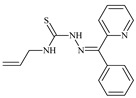 **	18.7	≥100	40.2	≥100	7.9	[46,47,48,83]
**L^8^**	** 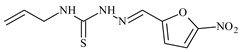 **	22.1	≥100	≥100	≥100	≥100	[46,47,48,83]
**L^9^**	** 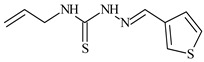 **	17.5	≥100	≥100	≥100	≥100	[46,47,48,83]
**L^10^**	** 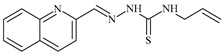 **	≥100	≥100	≥100	≥100	≥100	[46,47,48]
**L^12^**	** 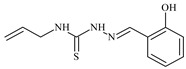 **	13.8	≥100	≥100	≥100	≥100	[46,47,48,83]
**L^13^**	** 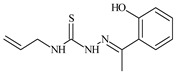 **	92.8	≥100	≥100	12	≥100	[46,81,83]
**L^14^**	** 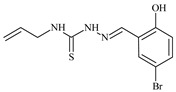 **	8.5	≥100	≥100	≥100	≥100	[46,47,48,81,83]
**L^15^**	** 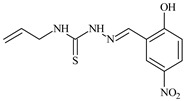 **	8.0	36.5	≥100	≥100	≥100	[46,47,48,70,74,83]
**L^16^**	** 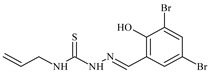 **	11.9	≥100	≥100	≥100	≥100	[46,47,48,70,74,81]
**L^17^**	** 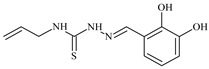 **	5.7	≥100	≥100	≥100	≥100	[46,47,48,70,74,83]
**L^18^**	** 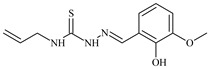 **	20.0	≥100	≥100	≥100	≥100	[46,47,48,70,81,83]
**L^19^**	** 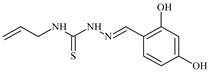 **	5.5	≥100	≥100	≥100	≥100	[46,47,48,70,81,83]
**L^20^**	** 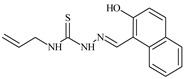 **	13.6	≥100	≥100	≥100	≥100	[46,47,48,74,81]
**L^21^**	** 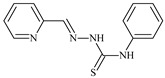 **	14.9	≥100	1.1	8.3	≥100	[47,48,49]
**L^22^**	** 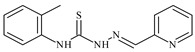 **	11.2	≥100	≥100	≥100	≥100	[47,48,49]
**L^23^**	** 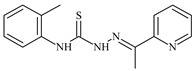 **	10.7	≥100	≥100	≥100	≥100	[47,48,49]
**L^24^**	** 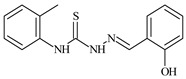 **	9.3	≥100	≥100	≥100	≥100	[47,48,49]
**L^25^**	** 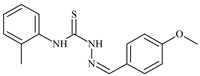 **	13.0	≥100	≥100	≥100	≥100	[47,48,49]
**L^26^**	** 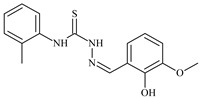 **	15.2	≥100	≥100	≥100	≥100	[47,48,49]
**L^27^**	** 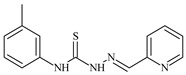 **	12.3	≥100	≥100	≥100	≥100	[47,48,49]
**L^28^**	** 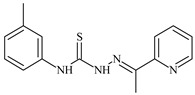 **	5.1	≥100	≥100	≥100	≥100	[47,48,49]
**L^29^**	** 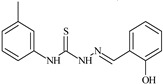 **	10.0	≥100	≥100	≥100	≥100	[47,48,49]
**L^30^**	** 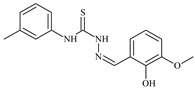 **	10.0	≥100	≥100	≥100	≥100	[47,48,72]
**L^31^**	** 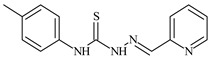 **	9.3	≥100	≥100	≥100	≥100	[47,48,72]
**L^32^**	** 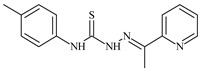 **	17.8	≥100	≥100	≥100	≥100	[47,48,72]
**L^33^**	** 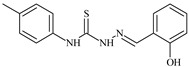 **	8.6	≥100	≥100	≥100	≥100	[47,48,72]
**L^34^**	** 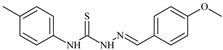 **	9.3	≥100	≥100	≥100	≥100	[47,48,72]
**L^35^**	** 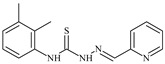 **	8.7 ± 0.1	≥100	≥100	≥100	≥100	[47,48,72]
**L^36^**	** 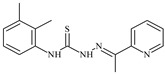 **	15.6	≥100	≥100	≥100	≥100	[47,48,72]
**L^37^**	** 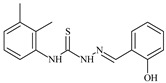 **	7.8	≥100	≥100	≥100	≥100	[47,48,72]
**L^38^**	** 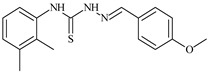 **	9.7	≥100	≥100	≥100	≥100	[47,48]
**L^39^**	** 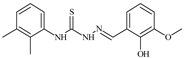 **	10.1	≥100	≥100	≥100	≥100	[47,48]
**L^40^**	** 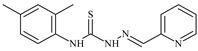 **	7.2	≥100	≥100	≥100	≥100	[47,48]
**L^41^**	** 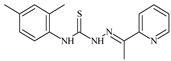 **	9.1	≥100	≥100	≥100	≥100	[47,48]
**L^42^**	** 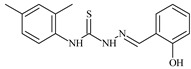 **	10.2	≥100	≥100	≥100	≥100	[47,48]
**L^43^**	** 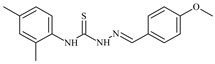 **	10.0	≥100	≥100	≥100	≥100	[47,48]
**L^44^**	** 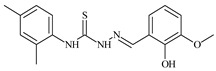 **	11.2	≥100	≥100	≥100	≥100	[47,48]
**L^45^**	** 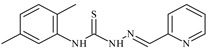 **	9.4	≥100	≥100	≥100	≥100	[47,48]
**L^46^**	** 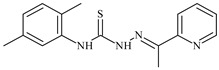 **	17.7	≥100	≥100	≥100	≥100	[47,48]
**L^47^**	** 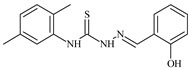 **	9.8	≥100	≥100	≥100	≥100	[47,48]
**L^48^**	** 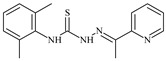 **	19.9	≥100	≥100	≥100	≥100	[47,48]
**L^49^**	** 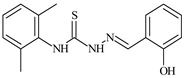 **	12.8	≥100	≥100	≥100	≥100	[47,48]
**L^50^**	** 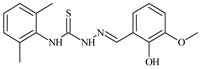 **	13.1	≥100	≥100	≥100	≥100	[47,48]
**L^51^**	** 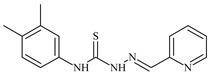 **	10.3	≥100	≥100	≥100	≥100	[47,48]
**L^52^**	** 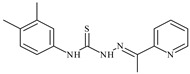 **	15.8	≥100	≥100	≥100	≥100	[47,48]
**L^53^**	** 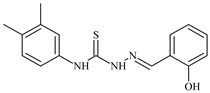 **	8.6	≥100	≥100	≥100	≥100	[47,48]
**L^54^**	** 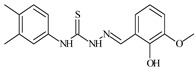 **	10.6	≥100	≥100	7.5	≥100	[47,48]
**L^55^**	** 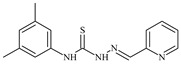 **	12.3	≥100	≥100	≥100	≥100	[47,48]
**L^56^**	** 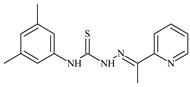 **	22.1	≥100	≥100	≥100	≥100	[47,48]
**L^57^**	** 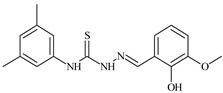 **	7.2	6.6	≥100	21.5	≥100	[47,48]
**L^58^**	** 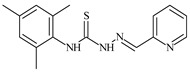 **	18.2	≥100	≥100	≥100	≥100	[47,48]
**L^59^**	** 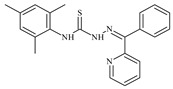 **	8.9	≥100	≥100	≥100	≥100	[47,48]
**L^60^**	** 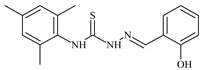 **	11.9	≥100	≥100	≥100	≥100	[47,48]
**L^61^**	** 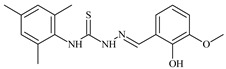 **	11.8	≥100	≥100	≥100	≥100	[47,48]
**L^62^**	** 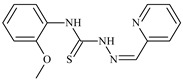 **	6.3	0.2	23.4	≥100	18.2	[47,48,49]
**L^63^**	** 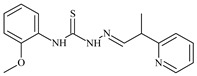 **	-	≥100	62.6	52.2	8.5	[47,48,49]
**L^64^**	** 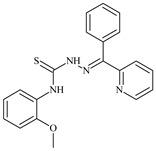 **	-	≥100	≥100	≥100	≥100	[47,48,49]
**L^66^**	** 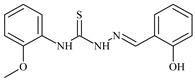 **	-	≥100	≥100	10.7	≥100	[47,48,49]
**L^67^**	** 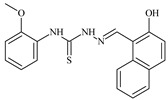 **	-	≥100	≥100	≥100	≥100	[47,48,49]
**L^69^**	** 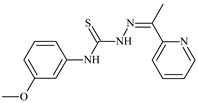 **	-	≤0.1	11.6	5.8	14.9	[47,48,49]
**L^70^**	** 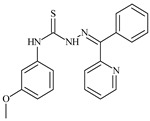 **	-	≥100	≥100	≥100	≥100	[47,48,49]
**L^71^**	** 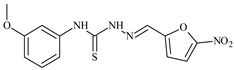 **	-	≥100	≥100	≥100	≥100	[47,48,49]
**L^72^**	** 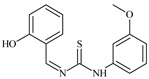 **	-	≥100	≥100	≥100	≥100	[47,48,49]
**L^73^**	** 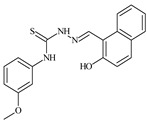 **	-	≥100	≥100	≥100	≥100	[47,48,49]
**L^74^**	** 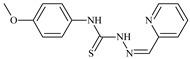 **	-	0.9	≥100	≥100	48.7	[47,48,49]
**L^75^**	** 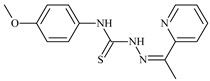 **	-	≤0.1	11.2	12.3	4.4	[47,48,49]
**L^76^**	** 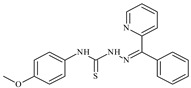 **	-	≥100	≥100	≥100	≥100	[47,48,49]
**L^77^**	** 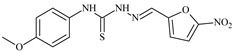 **	15.1	≥100	≥100	≥100	≥100	[47,48,49]
**L^78^**	** 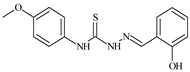 **	-	≥100	≥100	≥100	≥100	[47,48,49]
**L^79^**	** 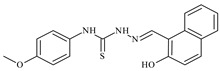 **	-	≥100	≥100	≥100	≥100	[47,48,49]
**L^80^**	** 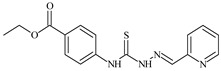 **	14.3	≥100	≥100	≥100	≥100	[47,48,84]
**L^81^**	** 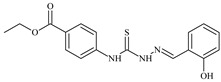 **	16.7	≥100	≥100	≥100	≥100	[47,48,84]
**L^82^**	** 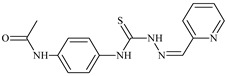 **	13.4	≥100	≥100	≥100	≥100	[47,48,84]
**L^83^**	** 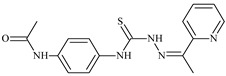 **	8.5	75.6	11.5	33.6	17.9	[47,48,84]
**L^84^**	** 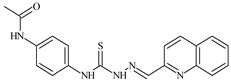 **	13.1	≥100	≥100	≥100	≥100	[47,48,84]
**L^85^**	** 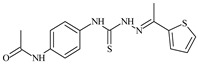 **	14.6	≥100	≥100	≥100	≥100	[47,48,84]
**L^86^**	** 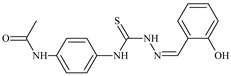 **	10.6	≥100	≥100	≥100	≥100	[47,48,84]
**L^87^**	** 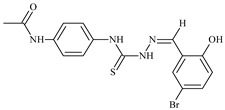 **	14.9	≥100	≥100	≥100	≥100	[47,48,84]
**L^88^**	** 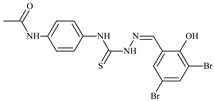 **	17.4	≥100	≥100	≥100	≥100	[47,48,84]
**L^89^**	** 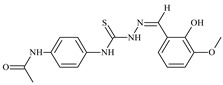 **	15.8	≥100	≥100	≥100	≥100	[47,48,84]
**L^90^**	** 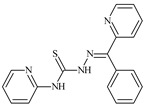 **	11.3	≥100	10.6	≥100	99.0	[47,48,84]
**L^91^**	** 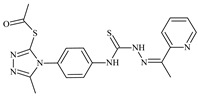 **	11.0	70.1	≥100	78.8	≥100	[47,48,84]
**L^92^**	** 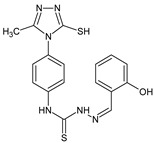 **	15.1	30.2	≥100	68.5	≥100	[47,48,84]
**L^93^**	** 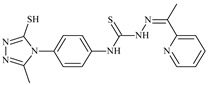 **	7.0	≥100	≥100	≥100	≥100	[47,48,84]

Average results of three experiments, SEM < ±3%.

The high antioxidant activity of these thiosemicarbazone ligands can be attributed to specific structural features, including the position and nature of functional groups and the molecular geometry [60]. Key structural factors contributing to high antioxidant activity include the presence of hydroxyl groups, as seen in 2-(2,3-dihydroxybenzylidene)-*N*-(prop-2-en-1-yl)hydrazinecarbothioamide and 2-(2,4-dihydroxybenzylidene)-*N*-(prop-2-en-1-yl)hydrazinecarbothioamide, which significantly enhance AOA by donating hydrogen atoms to neutralize free radicals. Their ortho or para positions facilitate stable electron density delocalization across the aromatic ring [57].

Alkenyl groups, such as those in 2-(2,4-dihydroxybenzylidene)-*N*-(prop-2-en-1-yl)hydrazinecarbothioamide, can enhance the overall reactivity of molecules, improving interactions with free radicals.

The combination of electron-accepting and -donating groups, like methoxy and methyl groups in *N*-(2-methoxyphenyl)-2-(pyridin-2-ylmethylidene)hydrazinecarbothioamide and *N*-(3-methylphenyl), stimulate the system by enhancing the molecule’s ability to retain and transfer electrons. Stabilization through hydrogen bonds, with amide and thiocarbamoyl groups forming additional hydrogen bonds as seen in *N*-[4-({[(2-(2-hydroxybenzylidene)hydrazinyl]carbonothioyl}amino)phenyl]acetamide, creates additional structural stability and activity [61].

Thus, the high AOA in these molecules is due to a combination of favorable factors such as the presence and position of hydroxyl, methoxy, and other functional groups, along with their overall molecular geometry. These characteristics enable these compounds to efficiently neutralize free radicals and protect cells from oxidative stress [57].

The study of thiosemicarbazones highlights the relationship between AOA and APA. The structural features of these compounds, including the presence, position, and type of functional groups such as *hydroxyl*, *methoxy*, and *alkenyl* groups, play a crucial role in determining their biological activities. The ability of these compounds to neutralize free radicals directly impacts their potential in reducing oxidative stress, which is a contributing factor in cancer progression. Similarly, their antiproliferative effects are enhanced by structural aspects.

The findings suggest that the structural of thiosemicarbazones can lead to the development of more effective anticancer agents with dual activity. The established structure–activity relationships (SARs) provide valuable insights for future research, guiding the design of new compounds that possess both high antioxidant and antiproliferative properties.

### 2.3. Antiproliferative and Antioxidant Activities of Isothiosemicarbazones

Isothiosemicarbazones are a class of organic compounds formed by the reaction of isothiocyanates with hydrazines or their derivatives. They are structural isomers of thiosemicarbazones, containing the functional group >C=N-NH-C(S) with the nitrogen atom in a configuration different from that found in thiosemicarbazones. Isothiosemicarbazones can be represented by the general formula R_1_R_2_C=NNH-C(S)NH_2_, where R_1_ and R_2_ are organic substituents. The presence of the isothiocyanate group makes them highly reactive, allowing them to form additional chemical bonds. Isothiosemicarbazones also have the capability to participate in various chemical reactions, including nucleophilic additions and substitutions, due to the double bond between carbon and nitrogen atoms [62]. Similar to thiosemicarbazones, isothiosemicarbazones may possess antioxidant properties, helping protect cells from oxidative stress by neutralizing free radicals. Some isothiosemicarbazones possess potential as anticancer agents, capable of inhibiting cancer cell growth by inducing apoptosis or causing cell cycle arrest [57].

Thus, isothiosemicarbazones represent an important class of compounds characterized by distinctive chemical properties and significant biological activity. Their applications in medicine, particularly as antioxidants, anticancer agents, and antimicrobial agents, make them a focal point of ongoing research and exploration in organic chemistry and pharmacology [63].

The results regarding the antioxidant and antiproliferative activities of the tested isothiosemicarbazones, expressed in IC_50_ values, are presented in Table 3.

The isothiosemicarbazone ligands methyl *N*-prop-2-en-1-yl-*N*’-[pyridin-2-ylmethylidene]carbamohydrazonothioate, methyl *N*’-[phenyl(pyridin-2-yl)-methylidene]-*N*-prop-2-en-1-ylcarbamohydrazonothioate, methyl *N*’-[(2-hydroxy-3-methoxyphenyl)methylidene]-*N*-prop-2-en-1-ylcarbamohydrazonothioate, ethyl *N*-prop-2-en-1-yl-*N*’-[pyridin-2-ylmethylidene]carbamohydrazonothioate, ethyl *N*-prop-2-en-1-yl-*N*’-[1-(pyridin-2-yl)ethylidene]carbamohydrazonothioate, and phenyl *N*’-[(2-hydroxyphenyl)methylidene]-*N*-prop-2-en-1-ylcarbamohydrazonothioate demonstrated selective APA. It should be noted that the introduction of iodine hydroxide into the ligands ethyl *N*-prop-2-en-1-yl-*N*’-[1-(pyridin-2-yl)ethylidene]-carbamohydrazonothioate (**L^102^**), *N*’-[(2-hydroxy-3-methoxyphenyl)methylidene]-*N*-prop-2-en-1-ylcarbamohydrazonothioate prop-2-en-1-yl (**L^104^**), and *N*-prop-2-en-1-yl-*N*’-[1-(pyridin-2-yl)ethylidene]carbamohydrazonothioate (**L^106^**) significantly enhanced their antiproliferative activity against the tested cancer cells (Table 3) [48].

The isothiosemicarbazone ligand **L^102^·HI** exhibited the highest activity, with an IC_50_ of 0.01 µM against BxPC-3 and 0.7 µM against RD cancer cells. **L^104^·HI** showed IC_50_ of 0.4 µM against BxPC-3, 1.0 µM against RD, and 1.4 µM against HeLa cancer cells. Similarly, **L^106^·HI** displayed IC_50_ of 0.5 µM against BxPC-3, 1.2 µM against RD, and 1.5 µM against HeLa cancer cells (Table 3) [48]. These results indicate that these isothiosemicarbazones exceed the antiproliferative effects of both doxorubicin and cisplatin [64].

This enhanced activity is likely because iodine is a large, polarizable halogen that can form halogen bonds with biological targets. These interactions can stabilize ligand **L^106^·HI**, improving the binding affinity and specificity for cellular targets associated with cancer cell proliferation. The addition of iodine may increase the lipophilicity of the compounds, facilitating their penetration through cell membranes and allowing the ligands to reach intracellular targets more effectively. Iodine can also withdraw electron density from specific parts of the molecule, potentially activating or deactivating certain functional groups, thus enhancing the reactivity of the ligands or binding efficiency [65].

Therefore, the differences in antiproliferative and antioxidant activities between isothiocarbazones and thiosemicarbazones can be attributed to their structural features and mechanisms of action. Isothiocarbazones exhibit greater antiproliferative activity due to their structure, which allows for more effective interaction with cellular targets, such as enzymes or receptors. The presence of the isothiocyanate group enables these compounds to covalently bind to functional groups in proteins, thereby interfering with their function and inhibiting cell growth [66].

However, the activity of thiosemicarbazones changes when they are transformed into corresponding S-substituted isothiosemicarbazones. For example, in the case of 2-hydroxybenzaldehyde derivatives, the activity of the corresponding isothiosemicarbazones is 2–3 times lower than that of thiosemicarbazones and is not significantly affected by the nature of the substituent at the sulfur atom. In contrast, S-methyl and S-ethyl *N*4-allylisothiosemicarbazones derived from 2-hydroxy-3-methoxybenzaldehyde exhibit higher activity than their corresponding thiosemicarbazones [48].

On the other hand, *S*-methyl-substituted 4-allylisothiosemicarbazones of 2-formyl-, 2-acetyl-, and 2-benzoylpyridines show practically no antiradical activity. Despite this, thiosemicarbazones demonstrate significant antioxidant activity [47], which is linked to their ability to delocalize charge and stabilize free radicals due to the thiocarbamide group containing nitrogen and sulfur atoms [67]. Thiosemicarbazones actively interact with free radicals by donating hydrogen atoms and neutralizing them, making them effective protectors against oxidative stress [68].

Thus, while isothiocarbazones possess higher antiproliferative activity, thiosemicarbazones exhibit significant antioxidant properties [47]. These differences underscore the importance of structural characteristics in determining the biological activity of compounds and their potential applications in medical practice.

The study of isothiosemicarbazones highlights a significant relationship between their structural characteristics and both APA and AOA. The findings reveal that specific functional groups, such as *hydroxyl*, *methoxy*, and *halogen* substituents like iodine, play a crucial role in modulating the biological activities of these compounds. *Hydroxyl* and *methoxy* groups enhance AOA by stabilizing free radicals through hydrogen donation, while the isothiocyanate functionality enables covalent interactions with cellular targets, thereby increasing APA. The incorporation of iodine not only improves the lipophilicity of these compounds but also boosts their binding affinity to biological targets, further amplifying their APA and AOA.

### 2.4. Antiproliferative and Antioxidant Activities of the 3d Metal Coordination Compounds of Thiosemicarbazones and Isothiosemicarbazones

The comparative analysis of 3*d* metal coordination compounds of thiosemicarbazones and isothiosemicarbazones highlights the impact of different 3*d* metals on their antiproliferative and antioxidant activities [69]. The study shows that the coordination of these ligands to metal centers generally enhances their antiproliferative properties but tends to reduce their antioxidant capabilities. Notably, a substantial number of these coordination compounds demonstrated antiproliferative activities exceeding those of well-known drugs like doxorubicin and cisplatin [48].

The study of 3*d* metal coordination compounds reveals a specific order of APA, Cu(II) complexes ≥ Ni(II) complexes ≥ Zn(II) complexes ≥ Co(III) complexes = Fe(III) complexes ≥ Cr(III) complexes, and AOA, Cr(III) complexes ≥ Mn(II) complexes = Fe(III) complexes ≥ Co(III) complexes = Ni(II) complexes = Cu(II) complexes = Zn(II) complexes (Table 4) [70,71,72,73,74,75].

The variability in biological activity can be attributed to factors such as the inherent properties of metal ions, the structure of proligands, and the biochemical environments in which these compounds function. Each transition metal’s unique coordination chemistry, including coordination numbers and geometric arrangements, plays a crucial role in ligand binding affinities, thereby influencing biological activity. For instance, the geometric configurations in Cu(II) complexes may enhance interactions with biological targets. Moreover, the oxidation states and reactivity of metals like copper and nickel may activate biochemical pathways that enhance antiproliferative effects [48].

Ligand structure is influential in activity modulation. Specific functional groups in thiosemicarbazones can increase the coordination compound’s stability and reactivity, boosting antiproliferative activity. Ligands capable of electron delocalization can stabilize radicals or efficiently interact with reactive sites in cancer cells, enhancing both antioxidant and antiproliferative activities. Additionally, the ability of these metal coordination compounds to target specific cellular enzymes or receptors involved in cell signaling contributes to their antiproliferative activity, with copper complexes showing particular promise in inducing apoptosis in cancer cells [76,77].

Regarding antioxidant activity, 3*d* metal coordination compounds facilitate free radical neutralization. Metal ions like chromium and manganese enable redox reactions that raise the antioxidant potential of the ligand, enhancing its protection against oxidative stress. Differing metabolic and distribution pathways of these compounds further contribute to their overall effectiveness [48].

The observed ranking in antiproliferative and antioxidant properties demonstrates the complex interplay between metal ions, ligand structures, and biological mechanisms. Cu(II) and Ni(II) coordination compounds may exhibit superior efficacy due to favorable interactions and chemical properties, while other metals may not reach similar activity levels due to their intrinsic characteristics. Continued research in this area can improve the design of effective anticancer agents and therapeutic tactics [78,79,80].

Typically, metal coordination compounds with 2-hydroxybenzaldehyde derivatives display higher antiradical activity than those with 2-formylpyridine derivatives [67]. The transformation of thiosemicarbazones to S-substituted isothiosemicarbazones shows variable impacts; sometimes, coordination compounds with 4-allylisothiosemicarbazone outperform their *N*4-allylthiosemicarbazone counterparts, and vice versa. The influence of acid residues on antiradical properties also lacks a consistent pattern [81,82].

The coordination of thiosemicarbazones and their derivatives to Cu(II) often significantly enhances the anticancer properties against various cancer cell lines. Incorporating certain *N*-heteroaromatic bases into the inner sphere of Cu(II) coordination compounds can amplify these properties and improve selectivity. While Cu(II) coordination compounds are typically highly active, they often lack selectivity. Thus, anticancer research should not be limited to Cu(II) compounds, as promising activity and selectivity have also been observed in Ni(II), Zn(II), Co(III), and Fe(III) coordination compounds [68].

The comparative analysis of 3*d* metal coordination compounds of thiosemicarbazones and isothiosemicarbazones underscores the complex interplay between various metal ions and their impacts on APA and AOA. The findings indicate that while the coordination of these ligands to different metal centers generally enhances their antiproliferative properties, it tends to reduce their antioxidant capabilities. Notably, many of these coordination compounds exhibit antiproliferative activities that surpass those of established chemotherapeutics such as doxorubicin and cisplatin.

The oxidation state of metal plays a critical role in determining the stability of coordination compounds. Generally, higher oxidation states are associated with increased stability of metal complexes in biological environments. This stability is advantageous because it allows the complexes to circulate in the bloodstream for longer periods, enhancing their bioavailability and therapeutic potential. The enhanced stability can also facilitate better targeting of cancer cells while minimizing off-target effects.

## 3. Comparative Characterization of Antiproliferative Activity and Anticancer Selectivity of Thiosemicarbazones and Their 3*d* Metal Coordination Compounds

Understanding the comparative antiproliferative activity and selectivity of thiosemicarbazones and their 3*d* metal coordination compounds is essential for the development of effective cancer therapies. Examining the differences in activity profiles between thiosemicarbazones and their respective metal complexes underlines how metal coordination can change their biological activity. By examining these differences, we can better understand the therapeutic potential of these compounds and how selectively they target cancer cells. Additionally, this analysis will clarify how these complexes work, helping us to design new and more effective anticancer agents that take advantage of both organic ligands and metal ions.

### 3.1. Antiproliferative Activity and Anticancer Selectivity of Allyl Thiosemicarbazone Pyridine Derivatives and Their 3d Metal Coordination Compounds

**Figure 1:**
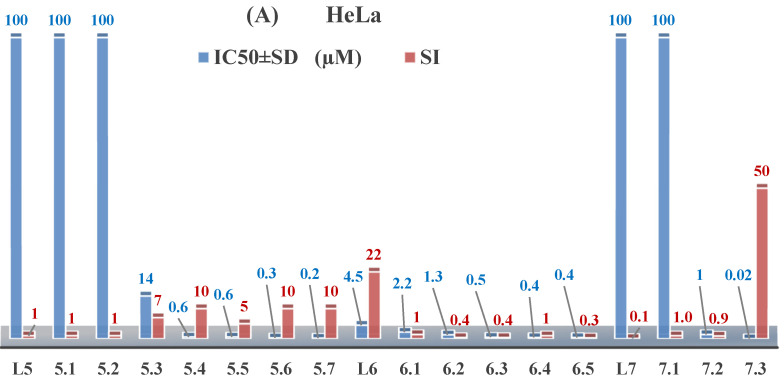

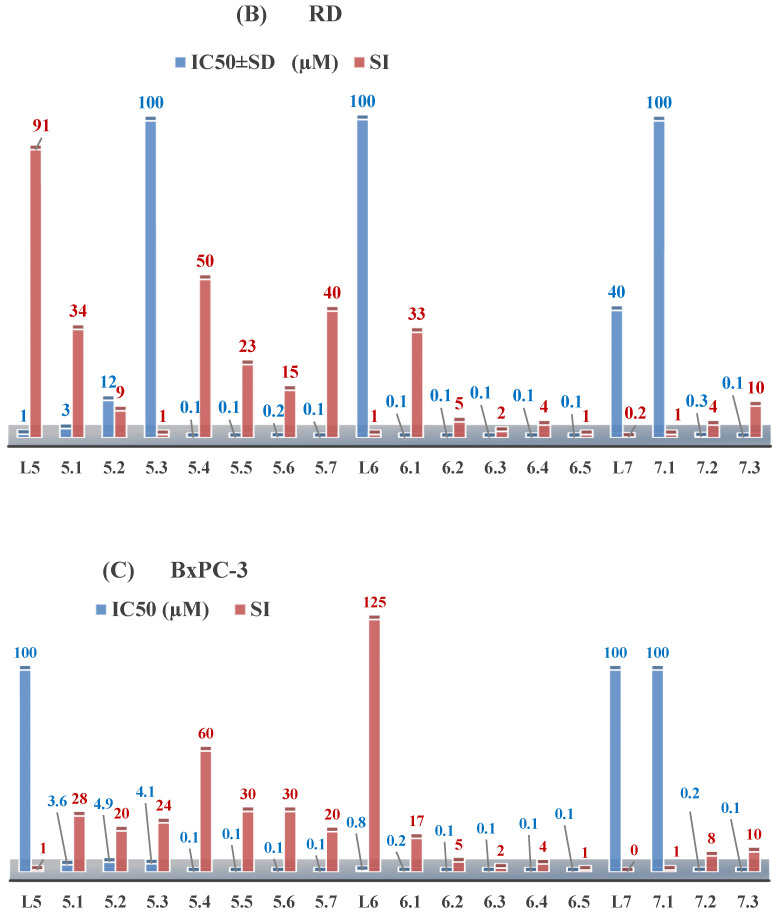
IC_50_ and SI of the synthesized coordination compounds of 3*d* metals with *N*-(prop-2-en-1-yl)-2-(pyridin-2-ylmethylidene)hydrazinecarbothioamide (**L^5^**), *N*-(prop-2-en-1-yl)-2-[1-(pyridin-2-yl)ethylidene]hydrazinecarbothioamide (**L^6^**), and 2-[phenyl(pyridin-2-yl)methylidene]-*N*-(prop-2-en-1-yl)hydrazinecarbothioamide (**L^7^**) towards the HeLa (**A**), RD (**B**), and BxPC-3 (**C**) cancer cell lines.

The ligands *N*-(prop-2-en-1-yl)-2-(pyridin-2-ylmethylidene)hydrazinecarbothioamide (**L^5^**), *N*-(prop-2-en-1-yl)-2-[1-(pyridin-2-yl)ethylidene]hydrazinecarbothioamide (**L^6^**), and 2-[phenyl(pyridin-2-yl)methylidene]-*N*-(prop-2-en-1-yl)hydrazinecarbothioamide (**L^7^**) showed that, with respect to the BxPC-3 and HeLa cancer cell lines, ligand **L^6^** exhibits the highest antiproliferative activity, with IC_50_ values of 0.8 and 4.5 µM, respectively, compared to **L^7^**, **L^5^**, as well as doxorubicin and cisplatin [67,83]. At the same time, **L^6^** has a high selectivity index (SI) of 125 for BxPC-3 and 22 for HeLa, indicating its lower toxicity to normal cells compared to cancerous ones. Ligand **L^5^** demonstrated APA against RD cells, with an IC_50_ of 1.1 μM and a selectivity index >90, but does not manifest any activity towards HeLa and BxPC-3 cell lines. The differences in activity between ligands **L^5^** and **L^6^** may be attributed to differences in structure, particularly in spatial configuration and types of functional groups, which affect their interactions with cellular targets (Figure 1) [68].

The functional groups present in these molecules are crucial for their antiproliferative activity. For instance, the pyridine ring, containing a nitrogen atom, facilitates coordination bonds with metal ions like copper and nickel, increasing the compounds’ electrophilicity. The presence of pyridine groups in **L^5^** and **L^6^** enhances the overall polarity, improving solubility and binding to biomolecules.

The allyl group (prop-2-en-1-yl) in both ligands **L^5^** and **L^6^** provides molecular flexibility, enabling interaction with various proteins and cellular targets. This flexibility may help the molecules adapt to different protein conformations, enhancing antiproliferative activity. Increased π-π interactions may also improve binding with target enzymes and receptors in cancer cells [84].

In compound **L^7^**, the phenyl group may increase lipophilicity and molecular volume, potentially hindering cell membrane penetration [68]. This can reduce overall interactions with cellular targets and diminish antiproliferative activity, explaining **L^7^**’s lower activity compared to that of **L^6^**.

As in the case of the copper(II) coordination compounds of **L^5^**, the introduction of *N*-heteroaromatic bases into the inner sphere in many cases increases the inhibitory effect of normal cells, reducing their selectivity indexes. It should be noted that all tested copper coordination compounds with proligand **L^5^**: **Cu(L^5^)(NO_3_)_2_**, **Cu(L^5^-H)(CH_3_COO)**, **Cu(L^5^-H)Cl, Cu(L^5^-H)Br**, and **(Cu(L^5^-H))_2_SO_4_**, exhibit activity against the tested cell lines, with IC_50_ ranging from 0.1 to 0.6 µM, which is significantly higher than the activity of the proligand. In contrast, coordination compounds **Co(L^5^-H)_2_Cl**, **Ni(L^5^-H)_2_**, and **Ni(L^5^)_2_Cl_2_** showed high activity only against BxPC-3, with IC_50_ of 4.1, 4.9, and 3.6 µM, respectively, while their activity against HeLa and RD cell lines was relatively low. In most cases, the activity of the coordination compounds, especially those containing copper, increases up to 1000 times compared to the ligand. Regarding selectivity, copper coordination compounds generally have higher selectivity [48].

The functional groups and radicals in the structure of ligand **L^5^** play a crucial role in determining the activity of its metal coordination compounds. The introduction of metals such as copper, cobalt, and nickel can significantly alter the electronic properties of the coordination compounds, influencing their binding with cellular targets. For instance, the presence of a pyridine ring in **L^5^** may facilitate the formation of coordination bonds with copper ions, enhancing antiproliferative activity by stabilizing the active configuration of the coordination compounds, which in turn affects its interaction with biomolecules [48].

Differences in the arrangement of functional groups, such as allyl and pyridine radicals, can also impact the ability of coordination compounds to interact with cancer cells. Copper coordination compounds exhibited higher biological activity compared to analogous nickel or cobalt coordination compounds due to the unique properties of the copper ion, such as its ability to participate in redox reactions and generate free radicals, which can promote apoptosis and inhibit cell growth [68].

Furthermore, the spatial arrangement of structural elements is also significant. For example, the planar configuration of some metal coordination compounds can enhance their interactions with DNA or other cellular structures, which explains their high activity against cancer cells. In contrast, bulkier or asymmetrical metal coordination compounds may hinder such interactions, potentially leading to reduced activity.

**L^6^** exhibits selective antiproliferative activity against BxPC-3 and HeLa cells with IC_50_ values of 0.8 and 4.5 µM, respectively, and demonstrated a high selectivity index. In the structure of **L^6^**, notable functional groups include the pyridine rings and the allyl group, which significantly influence its biological activity. The pyridine ring, containing nitrogen, enhances polarity and facilitates the ability of metal complexes to form coordination bonds with copper ions, which can increase its antiproliferative activity by binding to cellular targets such as DNA. The allyl group (prop-2-en-1-yl) provides structural flexibility, improving interactions with target proteins within the cell [48].

Copper coordination compounds such as **Cu(L^6^)(NO_3_)_2_**, **Cu(L^6^-H)(CH_3_COO)**, **Cu(L_6_-H)Cl, Cu(L_6_-H)Br**, and **(Cu(L_6_-H))_2_SO_4_** exhibit significantly greater antiproliferative activity. This is because copper, being a transition metal, greatly enhances covalent activity through the formation of stereospecific metal coordination compounds. The introduction of functional groups such as nitrate (NO_3_), acetate (CH_3_COO), and halogens (Cl and Br) also increases the electrophilicity of the metal complex, thereby enhancing its interaction with biomolecules and its ability to induce oxidative stress in cancer cells.

On the other hand, **L^7^** did not demonstrate antiproliferative activity against the cell lines, similar to its complex **Ni(L^7^)_2_(NO_3_)_2_**. The main distinction lies in the presence of the phenyl group in **L^7^**, which, compared to pyridine in **L^6^**, may increase the molecular volume, reducing its ability to penetrate the cell membrane. This limits its positive interactions with biological targets [48].

Nevertheless, copper coordination compounds **Cu(L^7^-H)(NO_3_)** and **Cu(L^7^-H)Cl** showed high activity against all cell lines, with IC_50_ values ranging from 0.02 to 1.40 µM. The inclusion of the pyridine fragment and the use of copper activates an antitumor mechanism through the enhancement of redox processes and the formation of free radicals, which is particularly critical for effectively targeting cancer cells.

Thus, the differences in functional groups and their spatial arrangement in the structures of compounds **L^6^** and **L^7^** significantly impact their antiproliferative activity. Pyridine groups, allyl, and halogen radicals interact in various ways, influencing both binding with biomolecules and the ability to penetrate cells. Copper, as a transition metal, enhances these interactions, whereas differences in the size and structure of functional groups can have both positive and negative effects on the biological activity of the compounds.

Specifically, the structures of ligands **L^6^** and **L^7^** feature functional groups that include a pyridine ring, which contains a nitrogen atom. This ring enhances the polarity of the molecule and facilitates the formation of coordination bonds with metal ions such as copper and nickel. The presence of pyridine groups increases the electrophilicity of the metal complex, promoting its binding with biomolecules and enhancing its antitumor activity [48].

The allyl group (prop-2-en-1-yl) is present in both **L^6^** and **L^7^** [68]. This functional group provides additional flexibility to the molecule and can improve interactions with proteins and other cellular targets, contributing to increased antiproliferative activity. In **L^7^**, a phenyl group is present, which increases the molecular size. This can reduce its ability to penetrate the cell membrane compared to **L^6^**, which in turn affects its antiproliferative activity.

Both molecules contain the hydrazinecarbothioamide functional group (R-NH-N=C(S)R), which grants them specific properties for interacting with biomolecules and cellular targets. This structure can promote binding with DNA and activate cellular signaling pathways.

In copper coordination compounds **(Cu(L^6^)(NO_3_)_2_**, **Cu(L^6^-H)(CH_3_COO)**, **Cu(L^6^-H)Cl**, **Cu(L^6^-H)Br**, and **(Cu(L^6^-H))_2_SO_4_)**, activity is enhanced due to the presence of copper ions. This facilitates the formation of reactive oxygen species, further enhancing their antiproliferative properties.

In copper coordination compounds with thiosemicarbazones that include NO_3_^−^ and CH_3_COO, these ligands can modify the electronic properties of the copper coordination compounds, influencing their reactivity and stability. Nitrate groups, in particular, can enhance the solubility of copper coordination compounds in aqueous environments, which is a crucial factor for their biological activity [48].

Therefore, the combination of these functional groups in thiosemicarbazone ligands and their copper coordination compounds significantly influences their pharmacological activity, selectivity, and toxicity. Copper coordination compounds, due to their ability to activate various mechanisms of action, exhibit high anticancer activity, but they may also display toxicity toward normal cells, related to their structural and functional properties.

**Figure 2:**
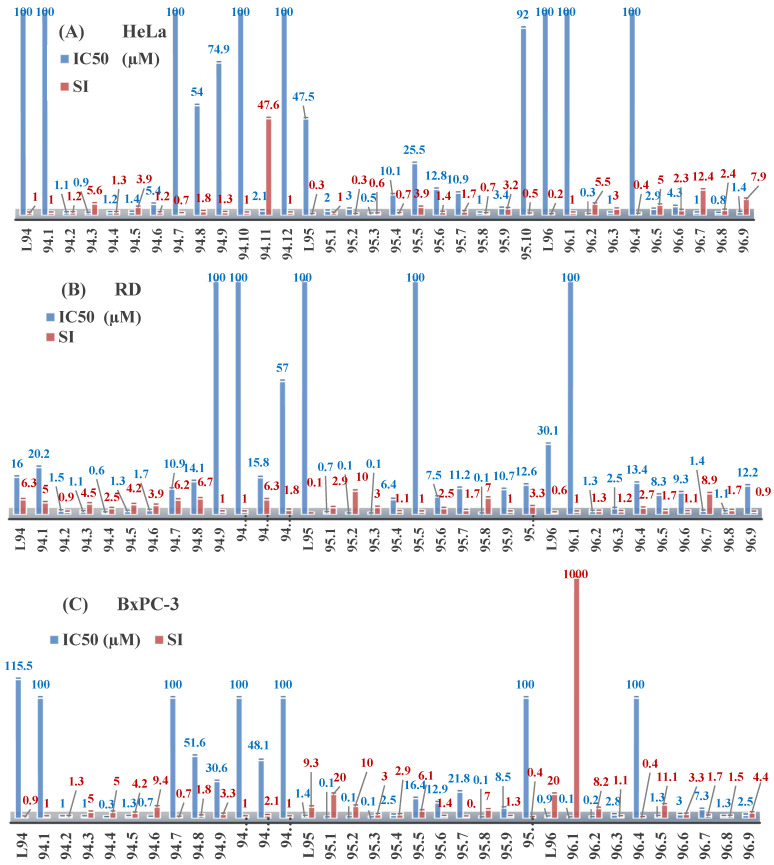
IC_50_ values and SI of the synthesized coordination compounds of 3*d* metals with methyl *N*-prop-2-en-1-yl-*N*’-[pyridin-2-ylmethylidene]carbamohydrazonothioate (**L^94^**), methyl *N*-prop-2-en-1-yl-*N*’-[1-(pyridin-2-yl)ethylidene]carbamohydrazonothioate (**L^95^**), and methyl *N*’-[phenyl(pyridin-2-yl)methylidene]-*N*-prop-2-en-1-ylcarbamohydrazonothioate (**L^96^**) towards the HeLa (**A**), RD (**B**), and BxPC-3 (**C**) cancer cell lines.

The antiproliferative activities of the compounds methyl *N*-prop-2-en-1-yl-*N*’-[pyridin-2-ylmethylidene]carbamohydrazonothioate (**L^94^**), methyl *N*-prop-2-en-1-yl-*N*’-[1-(pyridin-2-yl)-ethylidene]carbamohydrazonothioate (**L^95^**), and methyl *N*’-[phenyl(pyridin-2-yl)-methylidene]-*N*-prop-2-en-1-ylcarbamohydrazonothioate (**L^96^**) were investigated against cancer cells [48], as shown in Figure 2.

**L^95^** and **L^96^** have higher activity compared to **L^94^**, with IC_50_ values of 1.4 µM and 0.9 µM, respectively, against BxPC-3 cells. This can be attributed to the fact that **L^95^** and **L^96^** contain modified functional groups that enhance their reactivity and interaction with cellular targets. Specifically, **L^95^** has an added more reactive aliphatic group, while **L^95^** features a phenyl group that may improve binding to cellular receptors and amplify the antiproliferative effect [48].

On the other hand, all ligands exhibited low APA against the RD and HeLa cell lines, possibly due to the presence of various mechanisms of resistance and metabolism in these lines, which limit the effectiveness of the compounds against their specific targets.

Interestingly, **L^94^·HI** displayed properties similar to **L^94^**, while the addition of the HI group to **L^96^** resulted in a nine-fold enhancement of APA against BxPC-3, with a 50 times increased selectivity index, making **L^96^·HI** particularly promising, with a selectivity index of 1000. This indicates that the modification of **L^96^** with the HI group improves both its effectiveness against cancer cells and its selectivity, which is an important aspect in the development of new anticancer drugs.

All copper coordination compounds of thiosemicarbazone ligands (**L^94–96^**), as well as cobalt coordination compounds with ligand **L^94^**, showed enhanced APA against all tested cancer cell lines. However, the selectivity remained low, which renders these compounds less promising. The cobalt complex with ligand **L^94^** exhibited weak antiproliferative activity. The nickel coordination compounds **Ni(L^96^)_2_(NO_3_)_2_ and Ni(L^96^)_2_(ClO_4_)_2_** were found to be more active than the nickel coordination compounds of **L^94^** and **L^95^**. Thus, the structural modifications of thiosemicarbazones and their coordination compounds with transition metals significantly influence their antiproliferative activity and selectivity [48].

### 3.2. Antiproliferative Activity and Anticancer Selectivity of Aromatic Alil Thiosemicarbazones and Their 3d Metal Coordination Compounds

**Figure 3:**
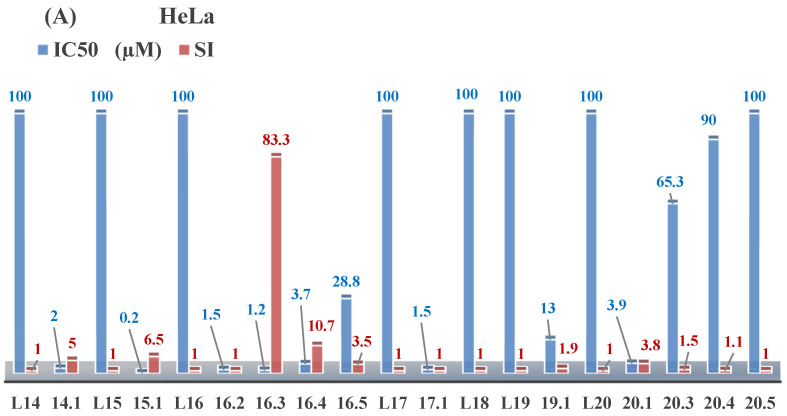

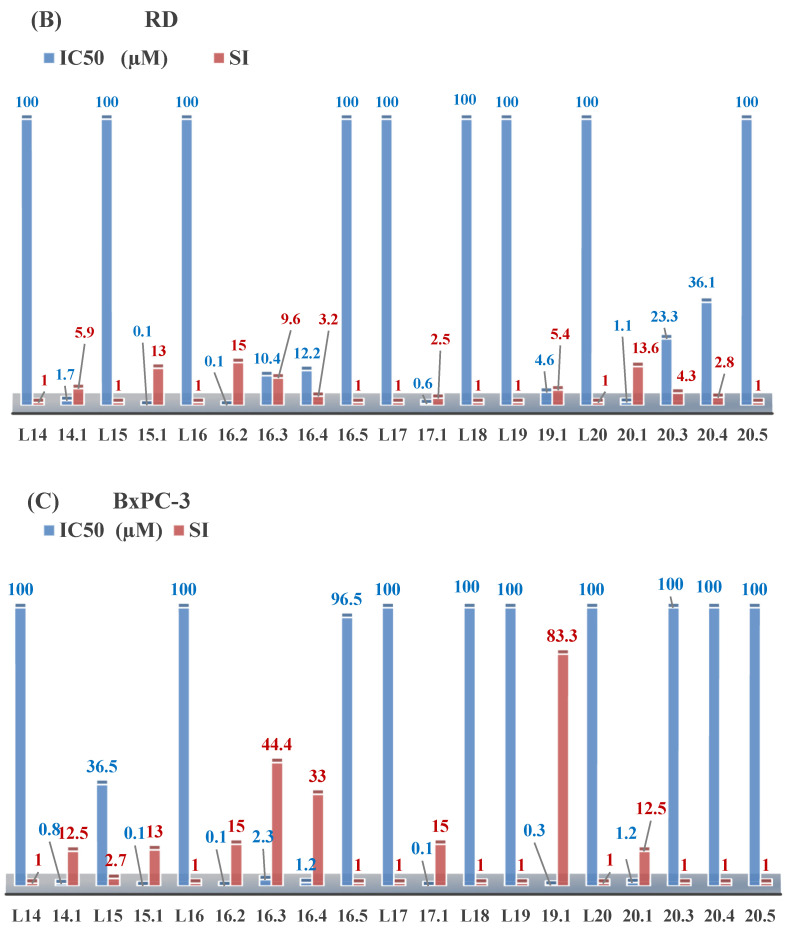
IC_50_ and SI of the synthesized coordination compounds of 3*d* metals with 2-(5-bromo-2-hydroxybenzylidene)-*N*-(prop-2-en-1-yl)hydrazinecarbothioamide (**L^14^**), 2-(2-hydroxy-5-nitrobenzylidene)-*N*-(prop-2-en-1-yl)hydrazinecarbothioamide (**L^15^**), 2-(3,5-dibromo-2-hydroxybenzylidene)-*N*-(prop-2-en-1-yl)hydrazinecarbothioamide (**L^16^**), 2-(2,3-dihydroxybenzylidene)-*N*-(prop-2-en-1-yl)hydrazinecarbothioamide (**L^17^**), 2-(2-hydroxy-3-methoxybenzylidene)-*N*-(prop-2-en-1-yl)hydrazinecarbothioamide (**L^18^**), 2-(2,4-dihydroxybenzylidene)-*N*-(prop-2-en-1-yl)hydrazinecarbothioamide (**L^19^**), and 2-[(2-hydroxynaphthalen-1-yl)methylidene]-*N*-(prop-2-en-1-yl)hydrazinecarbothioamide (**L^20^**) towards the HeLa (**A**), RD (**B**), and BxPC-3 (**C**) cancer cell lines.

Aromatic alil thiosemicarbazones, such as 2-(5-bromo-2-hydroxybenzylidene)-*N*-(prop-2-en-1-yl)hydrazinecarbothioamide (**L^14^**), 2-(2-hydroxy-5-nitrobenzylidene)-*N*-(prop-2-en-1-yl)hydrazinecarbothioamide (**L^15^**), 2-(3,5-dibromo-2-hydroxybenzylidene)-*N*-(prop-2-en-1-yl)hydrazinecarbothioamide (**L^16^**), 2-(2,3-dihydroxybenzylidene)-*N*-(prop-2-en-1-yl)hydrazinecarbothioamide (**L^17^**), 2-(2-hydroxy-3-methoxybenzylidene)-*N*-(prop-2-en-1-yl)-hydrazinecarbothioamide (**L^18^**), 2-(2,4-dihydroxybenzylidene)-*N*-(prop-2-en-1-yl)hydrazinecarbothioamide (**L^19^**), and 2-[(2-hydroxynaphthalen-1-yl)methylidene]-*N*-(prop-2-en-1-yl)hydrazinecarbothioamide (**L^20^**), demonstrate high antioxidant activity but did not exhibit significant antiproliferative activity against the HeLa, RD, BxPC-3 cell lines (Figure 3) [48].

The lack of antiproliferative activity can be attributed to several structural and functional characteristics. Although hydroxyl groups can facilitate the formation of hydrogen bonds and increase the polarity of the compounds, their presence alone may not be sufficient for effective binding with targets responsible for inhibiting cell proliferation. Hydroxyl groups may interact with polar molecules; however, their influence on antiproliferative activity may be limited [85]. The aromatic rings present in these compounds are capable of forming π-π interactions and stabilizing the structure, but this might not be enough to bind with key targets, such as enzymes or receptors, necessary to inhibit cancer growth [48].

Substituents such as bromine and nitro groups in some compounds, like **L^14^** and **L^15^**, may increase electronegativity and overall reactivity, but they do not always provide significant antiproliferative activity. Despite their electroactive properties, these groups may not exert the required effect on target molecules responsible for controlling cell proliferation. Substituents such as the methoxy group in **L^18^** can affect lipophilicity and solubility, but their impact on the interaction with cellular targets may also be limited. Together, these factors indicate that the functional groups and radicals in aromatic thiosemicarbazones can provide high antioxidant activity, yet their chemical structure and interaction with targets do not always result in significant antiproliferative activity [86].

Thus, while aromatic thiosemicarbazones demonstrate high antioxidant activity, their structure and mechanisms of interaction with cells may hinder the manifestation of an antiproliferative effect. At the same time, their copper (II) coordination compounds, such as **Cu(L^14^-H)Cl**, **Cu(L^15^-H)Cl**, **Cu(Py)(L^16^-H)NO_3_**, **Cu(L^16^-2H)H_2_O**, **Cu(L^16^-H)Br**, **Cu(L^17^-H)NO_3_**, **Cu(L^19^-H)NO_3_**, and **Cu(L^20^-H)Cl**, exhibit high antiproliferative activity, with IC_50_ values ranging from 0.1 to 10 µM. The selectivity index (SI) for these copper (II) coordination compounds, especially with respect to BxPC-3, ranges from 13 to 83, indicating their high selectivity. However, for the RD and HeLa cell lines, the selectivity index is less promising [48].

3*d* metal coordination compounds such as **Co(L^16^-H)_2_NO_3_**, **Co(L^20^-H)_2_NO_3_**, **Ni(L^20^)(L^20^-H)Cl**, and **Cr(L^20^-H)_2_NO_3_** did not demonstrate antiproliferative activity against the BxPC-3 cell line. Moreover, the complex **Cr(L^20^-H)_2_NO_3_** did not exhibit antiproliferative activity against any of the tested cell lines.

Copper coordination compounds, possessing the properties of transition metals, are capable of participating in redox reactions and generating reactive oxygen species, which can contribute to the induction of apoptosis in cancer cells. Furthermore, structural changes induced by the coordination of copper with thiosemicarbazones may enhance the binding of these compounds to cellular targets such as DNA and specific proteins responsible for regulating cell growth [87].

On the other hand, coordination compounds of cobalt, nickel, and chromium exhibited less APA. For instance, the presence of less reactive functional groups or an ineffective ability to form coordination bonds may limit their activity. In the case of chromium, its stable coordination compounds may not release the necessary active forms for modulating cellular functions, which explains the lack of antiproliferative activity [88].

Additionally, the influence of ligands on metal ions, and vice versa, results in a change in activity. Ligands containing hydroxyl and aromatic groups can affect the electron density of metals, altering their properties and reactivity. This can, in turn, enhance or diminish their antiproliferative properties. For example, strong electron donors like hydroxyl groups can increase the reactivity and stability of chelated metal coordination compounds, improving their interactions with biomolecules [89].

Metal ions can also impact the properties of ligands. During complex formation, metals can change the structure or spatial position of the functional groups in the ligand molecules, which can affect the ability of the ligand to bind to cellular targets or activate cellular signaling pathways. For instance, a metal may stabilize a certain conformation of the ligand, making it more suitable for interactions with target molecules [90,91].

The low selectivity for the RD and HeLa cell lines may indicate that these cells have certain survival mechanisms or resistance to reactive oxygen species, allowing them to withstand the effects of these metal coordination compounds. Overall, the interaction between structure, reactivity, and the mechanical properties of the compounds determined their biological activity and selectivity towards different cell lines.

**Figure 4:**
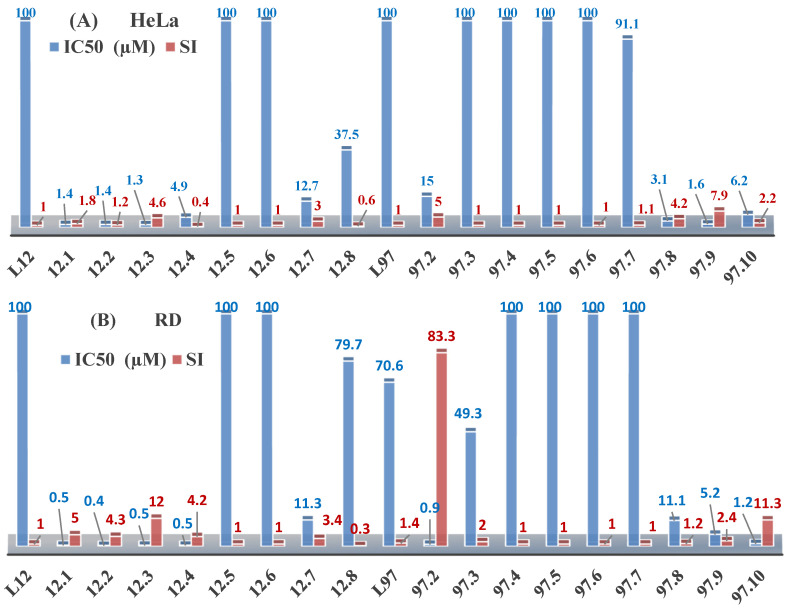

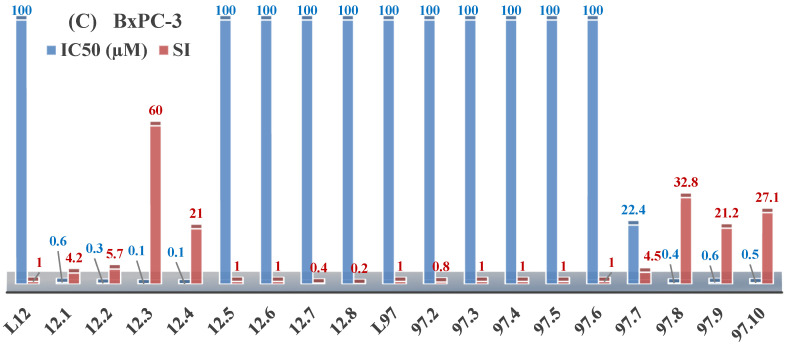
IC_50_ values and SI of the synthesized coordination compounds of 3*d* metals with (2*E*)-2-(2-hydroxybenzylidene)-*N*-(prop-2-en-1-yl)hydrazinecarbothioamide (**L^12^**) and methyl *N*’-[(2-hydroxyphenyl)methylidene]-*N*-prop-2-en-1-ylcarbamohydrazonothioate (**L^97^**) towards the HeLa (**A**), RD (**B**), and BxPC-3 (**C**) cancer cell lines.

The thiosemicarbazones, such as 2-(2-hydroxybenzylidene)-*N*-(prop-2-en-1-yl)hydrazinecarbothioamide (**L^12^**) and methyl *N*’-[(2-hydroxyphenyl)methylidene]-*N*-prop-2-en-1-ylcarbamohydrazonothioate (**L^97^**), did not exhibit significant antiproliferative activity against the tested cancer cell lines. However, their Cu (II) coordination compounds, including **Cu(L^12^-H)Cl**, **Cu(L^12^-H)Br**, **Cu(L^12^-H)(NO_3_)**, **Cu(L^12^-2H)H_2_O**, **Cu(L^97^-H)CH_3_COO**, **Cu(L^97^-H)Br**, and **Cu(L^97^-H)Cl**, demonstrated high antiproliferative activity across all cell lines. The highest activity was noted against the BxPC-3 line, with IC_50_ values ranging from 0.1 to 0.5 µM. Copper coordination compounds based on ligand **L^12^**, in particular, exhibited higher activity than those of ligand **L^97^**. Additionally, copper coordination compounds showed a higher selectivity index towards BxPC-3 cells, making them less toxic [48] (Figure 4).

Regarding **Zn(L^12^-H)Cl, Fe(L^12^-H)_2_(NO_3_)**, and **Fe(L^97^-H)_2_NO_3_**, these metal coordination compounds also exhibited significant activity across all cell lines. However, in some cases, their activity was an order of magnitude lower than that of the copper coordination compounds [92].

Cobalt and nickel coordination compounds, such as **Co(L^12^-H)_2_Cl**, **Ni(L^12^)(L^12^-H)Cl**, **Co(L^97^-H)_2_NO_3_**, **Co(L^97^-H)_2_Cl, Co(L^97^-H)_2_I**, **Ni(L^97^)(L^97^-H)ClO_4_**, and **Cr(L^97^-H)_2_NO_3_**, did not show activity against cancer cells, with IC_50_ values generally exceeding 100 µM.

These results can be explained by several factors. Copper coordination compounds, due to their unique redox properties and ability to generate ROS, can induce apoptosis in cancer cells. The high activity against BxPC-3 may be attributed to the specific characteristics of its cell membrane and metabolic pathways, making it more sensitive to active oxy-gen species [93].

On the other hand, cobalt and nickel coordination compounds may have lower reactivity or reduced capacity to form active oxygen species, which diminishes their potential antiproliferative activity. Additionally, the structural characteristics of the ligands and their substitutions may reduce binding with key cellular targets necessary for inhibiting the proliferation of cancer cells [94].

**Figure 5:**
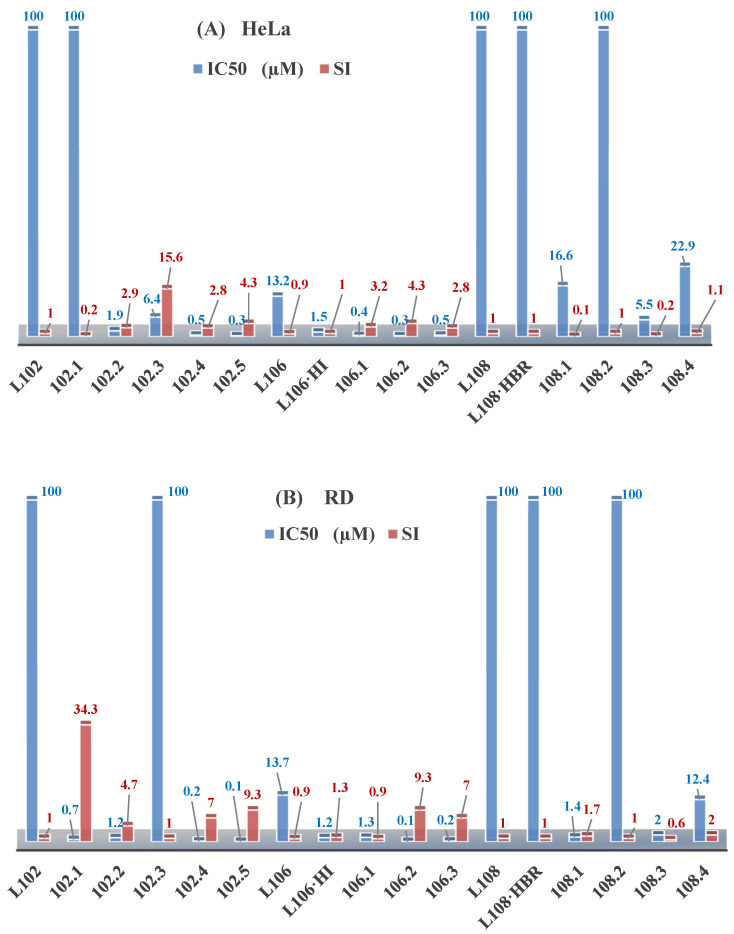

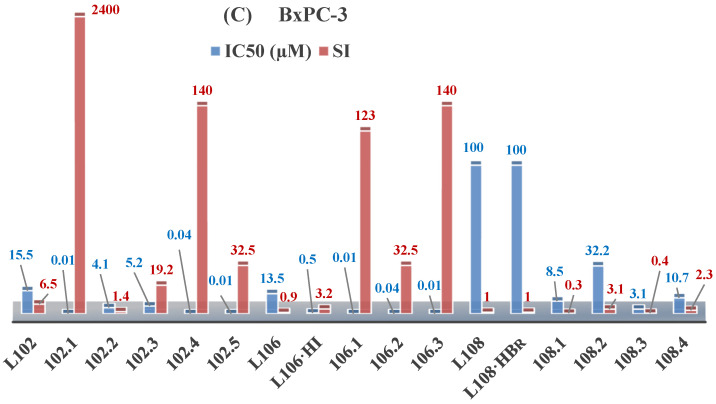
IC_50_ and SI of the synthesized coordination compounds of 3*d* metals with *N*-prop-2-en-1-yl-*N*’-[1-(pyridin-2-yl)ethylidene]carbamohydrazonothioate (**L^102^**), prop-2-en-1-yl *N*-prop-2-en-1-yl-*N*’-[1-(pyridin-2-yl)ethylidene]carbamohydrazonothioate (**L^106^**), (**L^102^·HI)**, 4-nitrobenzyl *N*-prop-2-en-1-yl-*N*’-[1-(pyridin-2-yl)ethylidene]carbamohydrazonothioate (**L^108^**), (**L^108^·HBr**) towards the HeLa (**A**), RD (**B**), and BxPC-3 (**C**) cancer cell lines.

The research on ethyl *N*-prop-2-en-1-yl-*N*’-[1-(pyridin-2-yl)ethylidene]carbamohydrazonothioate (**L^102^**) and prop-2-en-1-yl *N*-prop-2-en-1-yl-*N*’-[1-(pyridin-2-yl)ethylidene]carbamohydrazonothioate (**L^106^**) reveals their antiproliferative activities against the BxPC-3 cell lines, with IC_50_ of 15.5 µM and 13.5 µM, respectively. However, **L^106^** exhibited activity against both cancerous and normal cells, suggesting low selectivity and general toxicity, as illustrated in Figure 5. The inclusion of HI groups in the ligand structure increased its antiproliferative potency [48].

The coordination of ligand **L^106^** to copper significantly increased its activity, with enhancements up to 1350-fold and improved selectivity indices. The copper coordination compounds **Cu(L^106^)Br_2_**, **Cu(L^106^)(NO_3_)_2_**, and **Cu(L^106^)Cl_2_** exhibited IC_50_ of 0.01, 0.04, and 0.01 μM, respectively, for BxPC-3 cells, with selectivity indices of 123, 32, and 140 [48].

The increased antiproliferative activity is attributed to the central carbamohydrazonothioate fragment and alkene radicals, with HI groups enhancing molecular stabilization. Thus, the modification of radicals and the introduction of metals such as copper can significantly boost the effectiveness of antitumor agents [95].

Further studies showed that ligand **L^102^** initially lacked antiproliferative activity against RD and HeLa cell lines. However, the addition of an HI group significantly enhanced its activity, resulting in IC_50_ of 0.01 and 0.7 μM against BxPC-3 and RD cells, respectively, with selectivity indices of 2400 and 34. Coordination with copper also improved the activity of compounds **L^102^·HI**, with **Cu(L^102^)Cl_2_** and **Cu(L^102^)(NO_3_)_2_** displaying IC_50_ of 0.01 and 0.04 μM [48].

Research on 4-nitrobenzyl *N*-prop-2-en-1-yl-*N*’-[1-(pyridin-2-yl)ethylidene]carbamohydrazonothioate (**L^108^**) and its bromide form **L^108^·HBr** indicated no antiproliferative activity. However, the coordination of **L^108^** to copper significantly enhanced its bioactivity, emphasizing the impact of metal addition.

Cobalt, nickel, and iron coordination compounds exhibited significantly lower antiproliferative activity compared to their copper counterparts, indicating that copper is particularly effective in enhancing biological activity [96].

## 4. Action Mechanisms of the Antiproliferative and Antioxidant Activities of Some Thiosemicarbazones and Their 3*d* Metal Coordination Compounds

Many thiosemicarbazones can intercalate between DNA bases, leading to structural changes in the DNA helix. This intercalation can inhibit replication and transcription processes, which are critical for cell division and survival [97]. Metal coordination compounds can enhance this interaction through coordination with DNA, promoting breaks and damage to the genetic material. For example, copper(II) coordination compounds with thiosemicarbazones have been shown to be inhibitors of topoisomerase-IIα. This enzyme is essential for relieving torsional stress in DNA during replication. Inhibiting this enzyme can lead to the accumulation of stress in DNA, ultimately resulting in apoptosis of the cells [98,99].

Metal coordination compounds can also generate ROS through Fenton-like reactions, creating oxidative stress within cells. This oxidative stress can damage cellular components, including lipids, proteins, and nucleic acids, leading to apoptosis [100]. Thiosemicarbazones generally possess antioxidant properties, which can provide a dual action: promoting oxidative damage to cancer cells while protecting normal cells under controlled conditions [101]. These compounds can initiate intrinsic apoptotic pathways, which involve the release of cytochrome c from mitochondria and the activation of caspases. This results in programmed cell death, a key objective in chemotherapy treatment [102,103].

The enhanced activity against certain cancer cell lines, especially those with high expression of topoisomerase-IIα, suggests a high degree of selectivity for malignant cells compared to normal cells. This selectivity might be linked to the higher metabolic rates and unique signaling pathways in cancer cells, making them more vulnerable to these compounds [104].

Structural modifications of thiosemicarbazones, such as the introduction of substituents or changes in the coordination environment of metal coordination compounds, significantly impact their biological activity [105]. Substituents can increase lipophilicity, enhancing cell membrane permeability and bioavailability [106].

While some compounds may induce oxidative stress in cancer cells, they can also provide antioxidant activity, protecting normal cells from damage and thus increasing therapeutic efficacy [107].

The mechanisms of action of thiosemicarbazones and their metal coordination compounds are multifaceted, involving direct interactions with DNA, the inhibition of essential enzymes, the generation of reactive species, and the induction of apoptotic pathways. The specific action can vary greatly depending on the chemical structure of the compounds, their metal coordination, and the biological context of the target cells [108].

The effect of 2-formylpyridine *N*(4)-phenylthiosemicarbazone (**L^21^**), copper(II) complex **Cu(L^21^)Cl**, and mixed complex **Cu(Str)(L^21^)Cl** with 4-aminobenzenesulfonamide (Str) on cytoplasmic vacuolization preceding apoptosis was studied [109].

The ability of these compounds to induce cellular vacuoles and apoptotic bodies was evaluated using microscopy. Morphological changes in the MeW-164, HeLa, and RD cells after 24 h of treatment at a concentration of 10 μM indicated pronounced cytoplasmic vacuolization. All experiments demonstrated a concentration-dependent relationship between the inhibitory effects of the compounds. The complexes **Cu(L^21^)Cl** and **Cu(Str)(L^21^)Cl** exhibited higher antiproliferative activity compared to thiosemicarbazone, exceeding the activity of DOXO by seven times. The coordination of organic molecules with the metal center significantly enhances their antiproliferative properties, while thiosemicarbazone shows greater selectivity [110,111].

In addition to visualizing apoptotic bodies, the mechanism of action was investigated by assessing the effects on genomic DNA isolated from HeLa cells. Electrophoretic separation of the DNA indicated fragmentation after 24 h of treatment. The interaction of the compounds with DNA led to fragmentation, activating apoptosis. During this process, biochemical changes occur, including loss of membrane asymmetry, cell detachment, reduction in cell size, nuclear fragmentation, and chromatin condensation [112].

DNA fragmentation is characteristic of the later stages of apoptosis when calcium- and magnesium-dependent nucleases are activated to degrade genomic DNA. This confirms that the tested compounds induce apoptotic processes in HeLa cells through their interaction with genomic DNA and subsequent fragmentation. This is significant because programmed cell death is a primary target for chemotherapy, and the disruption of apoptotic pathways, common in cancer, poses a serious challenge to effective treatment [113].

The microscopic fluorescent method of flow cytometry using the NucleoCounter also confirmed the mechanism of apoptosis induced by these compounds.

2-Formylpyridine *N*(4)-allylthiosemicarbazone (**L^21^**) has been studied for its interactions with metal ions, specifically copper(II) and nickel(II) [68]. The coordination of **L^21^** to transition metals significantly alters the electronic properties of the compound, enhancing its antiproliferative activity. Notably, studies show that both copper(II) and nickel(II) complexes exhibit potent activity against HL-60 cancer cells, with copper coordination compounds being four times more active than the parent thiosemicarbazone [114].

Nuclear magnetic resonance (NMR) spectroscopy has determined that the energies of hydrogen bonds formed by the interaction of **L^21^** with guanine fragments in DNA range from 8 to 13 kJ/mol. The high electron density on the nitrogen atom of the pyridine ring facilitates hydrogen bond formation with DNA, triggering programmed cell death (apoptosis). Additionally, monomeric structures of the compounds allow for intercalation between the nitrogenous bases of nucleic acids.

Research by Zeglis B.M. et al. [115] has shown that both 2-formylpyridine *N*(4)-allylthiosemicarbazone and its copper(II) chloride complex inhibit topoisomerase-IIα at lower concentrations, which is critical for cell proliferation regulation. Copper coordination compounds demonstrating antiproliferative activity against breast cancer cells (SK-BR-3) with high expressions of topoisomerase-IIα are more effective than those against cells with lower levels of this enzyme (MCF-7) [83].

Magnetochemical studies reveal that the position of the carbonyl group influences the antiproliferative activity of 4-allylthiosemicarbazone [68]. A characteristic magnetic moment indicative of a single unpaired electron supports a monomeric structure, emphasizing its role in biological activity. Research indicates that monomeric compounds can intercalate between the nitrogenous bases of nucleic acids, potentially leading to apoptosis. *N*(4)-allylthiosemicarbazone also exhibited high antiproliferative activity against HL-60 cancer cells (IC_50_ = 0.3 µM). It was established that the high electron density on the nitrogen atom of the pyridine ring allows the molecule to form hydrogen bonds with DNA.

Using density functional theory (DFT) with Multiwfn software [116,117], the electronic structure and molecular electrostatic potential (MEP) maps [117] for **L^21^** were calculated. The MEP ranges from −31.7 kcal·mol^−1^ (red, indicating electrophilic sites) to +31.7 kcal·mol^−1^ (blue, indicating nucleophilic sites). The negative electrostatic potentials are primarily located around the nitrogen and sulfur atoms, while positive potentials are found at the ends of the CH and NH groups.

Thus, the interaction of these molecular inhibitors of cancer cell proliferation with DNA occurs through hydrogen bond formation, the initiation of programmed cell death, or apoptosis [118,119].

Notably, copper coordination compounds exhibit lower selectivity compared to the precursor **L^21^**, while nickel complexes demonstrate significantly higher selectivity. As such, the nickel coordination compound has been patented as a promising anticancer agent against the HL-60 cell line. Moreover, the introduction of specific *N*-heteroaromatic bases into the inner sphere of copper coordination compounds has been shown to enhance their inhibitory activity toward normal cells.

Thus, an expanded understanding of the mechanisms underlying the antiproliferative activity of thiosemicarbazones and their 3*d* metal coordination compounds reveals a multifaceted approach to combating cancer cells. These compounds can intercalate between DNA bases, leading to structural changes that impede replication and transcription—processes vital for cell division and survival. Metal coordination compounds, particularly those formed with copper(II), enhance these effects. They inhibit enzymes such as topoisomerase-IIα, essential for managing DNA torsional stress during replication, and catalyze Fenton-like reactions that generate ROS, promoting oxidative damage and apoptosis in cancer cells.

Beyond their antiproliferative effects, thiosemicarbazones demonstrate significant antioxidant activity. These compounds can reduce oxidative stress not only by inducing oxidative damage in cancer cells but also by exerting a protective effect on normal cells due to their inherent antioxidant properties. This dual capability is reflected in their role in modulating cellular redox states; while they selectively promote oxidative stress in malignant cells, they can simultaneously stabilize the redox balance in healthy cells. The presence of substituents that increase lipophilicity and permeability through cell membranes further enhances both antiproliferative and antioxidant efficacy by improving bioavailability and interaction with cellular targets.

The structural adaptability of thiosemicarbazones, including modifications that introduce specific functional groups, impacts their ability to manage oxidative stress and apoptosis pathways. Their antioxidant action comes to the fore in the context of exploiting the oxidative environment of cancer cells, thereby turning a typical vulnerability into a targeted therapeutic approach. This capacity to manage oxidative environments positions thiosemicarbazones and their metal complexes as bifunctional agents in cancer therapy, simultaneously tackling cancer cell proliferation and protecting normal cells from oxidative damage. The therapeutic implications are profound, warranting further exploration of their antioxidant mechanisms alongside their antiproliferative effects.

## 5. Conclusions

Thiosemicarbazone-based compounds have garnered significant interest in recent years due to their remarkable potential as inhibitors of cancer cell proliferation. These compounds not only exhibit powerful antiproliferative effects but also possess antioxidant properties, which are crucial in combating oxidative stress associated with cancer progression. This review discusses the dual role of thiosemicarbazone-based compounds as therapeutic agents, focusing on their mechanisms of action and implications in cancer treatment.

We examined in detail their antioxidant activity and antiproliferative activity, as well as how the antitumor activity is related to the antioxidant properties of thiosemicarbazones, isothiosemicarbazones, and their coordination complexes with transition metals such as zinc(II), copper(II), nickel(II), cobalt(III), and iron(III). We also considered the selectivity of these compounds.

Research indicates that coordinating thiosemicarbazones with copper(II) enhances their antiproliferative properties; however, these compounds often lack optimal selectivity, though their selectivity indexes compare favorably with those of doxorubicin and cisplatin used in medical practice. Notably, copper coordination compounds derived from *N*4-allylthiosemicarbazide have shown significant inhibitory effects against various cancer cell lines, often surpassing established chemotherapeutic agents. Interestingly, coordination compounds involving nickel(II), iron(III), and cobalt(III) may exhibit lower overall activity against cancer cells but demonstrate improved selectivity, targeting cancer cells more effectively than normal cells. This emphasizes the need to explore a broader array of transition metal coordination compounds for developing selective molecular inhibitors against various cancer cell lines.

Furthermore, enhancing the anticancer properties and selectivity of copper(II) coordination compounds can be achieved by introducing specific *N*-heteroaromatic bases. The search for new anticancer agents should extend beyond copper(II) to include promising candidates from nickel(II), zinc(II), cobalt(III), and iron(III) coordination compounds, which possess noteworthy selectivity.

This study underscores the importance of the antioxidant activity of thiosemicarbazones and isothiosemicarbazones, considering the role of oxidative stress in cancer progression. This work presents data on the ability of these compounds to neutralize free radicals, which is critical for protecting cellular components from oxidative damage. The results indicate that certain coordination compounds, especially those containing nickel, zinc, and iron, demonstrate superior antioxidant activity compared to their copper and cobalt counterparts. Notably, it was observed that many coordination compounds generally exhibit lower antioxidant activity than their corresponding free ligands.

Electrophoretic separation techniques revealed that interactions between the tested compounds and DNA resulted in fragmentation, highlighting the initiation of apoptosis in these cells. This apoptosis was characterized by significant morphological changes and was further corroborated through a microscopic cell analysis and flow cytometry.

Furthermore, an X-ray diffraction analysis revealed the electron density distribution within one of the most promising molecules, 4-allylthiosemicarbazone 2-formylpyridine, which exhibits high antiproliferative activity. The data indicate that a high electron density on the nitrogen atom of the pyridine ring plays a crucial role in facilitating the formation of hydrogen bonds with DNA. This interaction underscores the compound’s capacity to interfere with DNA functions.

Nuclear magnetic resonance (NMR) studies supported these findings, confirming that the interactions between the molecular proliferation inhibitors and DNA indeed lead to the establishment of hydrogen bonds. Such interactions are pivotal for apoptotic signaling pathways, emphasizing that enhanced binding to genomic DNA is a significant mechanism underlying the anticancer activity of these compounds.

Our correlation analysis of the overall data block conducted as part of our study indicates a potential correlation between AOA and APA. This suggests that some compounds with high antioxidant activity may also have enhanced anticancer properties. We identified 80 metal coordination compounds and 10 thiosemicarbazone proligands that demonstrated higher APA than doxorubicin or cisplatin, as well as greater AOA compared to Trolox. This may occur due to similar or complementary mechanisms.

The observation that some compounds may operate through similar or complementary mechanisms highlights the need for further exploration of the relationships between AOA and APA. Investigating how antioxidant properties can enhance the anticancer efficacy of specific compounds is crucial for developing more potent therapeutic strategies.

This research illustrates that, despite extensive previous studies on thiosemicarbazones, many unknowns remain. Their coordination with 3*d* metal ions and modifications of their inner sphere can lead to the development of new compounds with promising biological activity and selective action. Therefore, these substances may have potential applications in drug development, targeted therapies, and combination treatments.

## 6. Future Research Directions

The thiosemicarbazones and isothiosemicarbazones discussed in this article show impressive anticancer activities, with potency levels reaching up to 600 times greater than doxorubicin and up to 1000 times greater than cisplatin. Their selectivity indices are noteworthy, reaching values of 1300 and 2400, respectively, making these compounds promising candidates for cancer treatment.

The research highlighted specific compounds that not only exhibit significantly higher antiproliferative activities but also demonstrate lower toxicity compared to traditional chemotherapy agents. This is important because it suggests that these compounds could provide better treatment options while reducing the side effects often associated with chemotherapy.

A detailed analysis of the SAR reveals that the unique structural features of these compounds play a crucial role in their enhanced effectiveness. Understanding which molecular characteristics contribute to improved activity will be key for future compound design.

The findings from this study emphasize the need for further exploration and development of these novel agents. By investigating their biological mechanisms and optimizing their structures, researchers can improve cancer treatment strategies, providing safer and more effective options for patients.

Despite substantial previous research on thiosemicarbazones and isothiosemicarbazones, the field still holds many unknowns and opportunities for discovery. Studying coordination chemistry with 3*d* metal ions and strategically modifying their inner structures may lead to new compounds with promising biological activities and selectivity.

Overall, exploring thiosemicarbazones and isothiosemicarbazones as innovative pharmacological agents against cancer could unlock their full potential, significantly enhancing cancer treatment protocols and improving patient survival rates.

## 7. Patents

As a result of the work reported in this manuscript on the antiproliferative activity of various compounds, four patents have been registered. These patents encompass innovative formulations and processes designed to enhance the effectiveness of the identified compounds against targeted cancer cell lines. The registered patents are anticipated to facilitate the development of new therapeutic agents with improved specificity and reduced side effects for cancer treatment. Ongoing research and development efforts are focused on optimizing these compounds for potential clinical applications while exploring their full therapeutic potential in oncology.

1. MD 4620 B1. BOPI nr. 2/2019. GULEA Aurelian, MD; ISTRATI Dorin, MD; ŢAPCOV Victor, MD; GARBUZ Olga, MD; GUDUMAC Valentin, MD; GROPPA Stanislav, MD. Use of di(µ-S)-bis{(4-aminobenzenesulfamide)-chloro-{N-[phenyl-2-(pyridine-2-ylmethylidene)hydrazine-1-carbothioamido(1-)]}}copper as a cancer cells proliferation inhibitor.
2. MD 4349 B1. BOPI nr. 5/2015. GULEA Aurelian, MD; LIPKOWSKI Andrzej, PL; GARBUZ Olga, MD; MATALINSKA Joanna, PL; ŢAPCOV Victor, MD. N-(3-methoxyphenyl)-2-(pyridine-2-ylmethylene)-hydrazinecarbothioamide compound—inhibitor of human melanoma MeW-164 cell proliferation.
3. MD 4778 B1. BOPI nr. 12/2021. GULEA Aurelian, MD; GRAUR Vasilii, MD; ŢAPCOV Victor, MD; GARBUZ Olga, MD; ANDRONACHE Lilia, MD; CEBAN Emil, MD; GUDUMAC Valentin, MD. Dichloro{methyl-N-(prop-2-en-1-yl)-2-[1-(pyridin-2-yl)ethylidene]hydrazinecarbimidothioate-N,N,N}copper compound, inhibiting the proliferation of human rhabdomyosarcoma cells.
4. MD 4764 B1. BOPI nr. 8/2021. GULEA Aurelian, MD; GRAUR Vasilii, MD; USATAIA Irina, MD; GARBUZ Olga, MD; ŢAPCOV Victor, MD. Dibromo{methyl-N-(prop-2-en-1-yl)-2-[1-(pyridin-2-yl)ethylidene] hydrazinecarbimidothioate-N,N,S}copper compound, inhibiting the proliferation of human rhabdomyosarcoma cells.
5. MD 4407, 3 2016. A. Gulea, V. Graur and V. Țapcov, “Inhibitor al celulelor HL-60 ale leucemiei umane mieloide în baza hidratului clorurii de bis[*N*-(prop-2-en-1-il)-2-(piridin-2-ilmetiliden)-hidrazincarbotioamid]-nichel(II)”.
6. MD 4698, BOPI 05/2020. GULEA Aurelian, MD; GUDUMAC Valentin, MD; ISTRATI Dorin, MD; USATAIA Irina, MD; GRAUR Vasilii, MD; ŢAPCOV Victor, MD; ŞVEŢ Inna, MD; PANTEA Valeriana, MD; Nitrat de catenă-(µ-nitrato-O,O“-O”)-{metil-N-(prop-2-en-1-il)-2-[1-(piridin-2-il)etiliden]hidrazincarbimidotioat}cupru(II) în calitate de inhibitor al radicalilor superoxizi.
7. MD 4755, BOPI 03/2021. GULEA Aurelian, MD; GUDUMAC Valentin, MD; ŢAPCOV Victor, MD; PANTEA Valeriana, MD; GRAUR Vasilii, MD; ANDRONACHE Lilia, MD; ŞVEŢ Inna, MD; BOTNARU Maria, MD; Compuşii coordinativi ai cuprului cu 4-aliltiosemicarbazonele 3- (fenil)-1-(piridin-2-il)prop-2-en-1-onelor substituite în calitate de inhibitori ai radicalilor superoxizi.
8. MD 4899, BOPI 08/2024, GULEA Aurelian, MD; ŢAPCOV Victor, MD; ISTRATI Dorin, MD; POIRIER Donald, CA; Utilizarea di(m-S)-bis{cloro-[N-(2,4-dimetilfenil)-N’-(piridin-2- ilmetiliden)carbamohidrazonotioat]cupru} tetrahidrat în calitate de inhibitor al proliferării celulelor HepG2 ale cancerului la ficat.
9. MD 4749, BOPI 03/2021. GULEA Aurelian, MD; GUDUMAC Valentin, MD; ISTRATI Dorin, MD; GRAUR Vasilii, MD; ŢAPCOV Victor, MD; PANTEA Valeriana, MD; ANDRONACHE Lilia, MD; ŞVEŢ Inna, MD; Utilizarea compuşilor coordinativi ai sărurilor de cupru(II) cu 2-(2- hidroxibenziliden)-N-(prop-2-en-1-il)-hidrazincarbotioamida în calitate de inhibitori ai radicalilor superoxizi.
10. Gulea Aurelian, Md., Ţapcov Victor, Md., Cebotari Diana, Md., Guţu Tatiana, Md., Istrati Dorin, Md., Gudumac Valentin, Md. [4-(2,4-Dimethylphenyl)-2-(2-hydroxy-3- methoxybenzylidene)hydrazinecarbothioamide-S][4-(2,4-dimethylphenyl)-2-, (oxo-3-methoxybenzylidene)hydrazinecarbothioamido(2-)-O,N,S]-nickel(II) monoethanol solvate as an antioxidant. 30 June 2019, BOPI nr. 6/2019.
11. Gulea Aurelian, Md., Usataia Irina, Md., Garbuz Olga, Md., Graur Vasilii, Md., Ţapcov Victor, Md., Gudumac Valentin, Md. Use of salicylidene-4-allyl-S-methylisothiosemicarbazidates of iron(III) and cobalt(III) as antioxidants. 30 November 2017, BOPI nr. 11/2017.
12. Gulea Aurelian, Md., Gudumac Valentin, Md., Garbuz Olga, Md., Ţapcov Victor, Md., Pahonţu Elena-Mihaela, Ro. Use of di(μ-S)-bis{(4-aminobenzenesulphamide)-chloro-[2-picolidene-4-phenylthiosemicarbazidato-(1-)]-copper(II)} as an antioxidant. 31 March 2017, BOPI nr. 3/2017.

## Figures and Tables

**Table 3:** Antiproliferative activity of isothiosemicarbazones against HeLa, RD, and BxPC-3 cancer cell lines, as well as the MDCK normal cell line. ABTS^•+^ scavenging activity of the compounds.

	Formula	ABTS IC_50_ (µM)	BxPC-3 IC_50_ (µM)	RD IC_50_ (µM)	HeLa IC_50_ (µM)	MDCK IC_50_ (µM)	Reference
**L^94^**	** 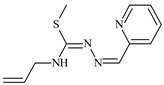 **	≥100	115.5	16.0	≥100	≥100	[46,47,48,67,73]
**L^94^·HI**		≥100	≥100	20.2	≥100	≥100	[46,47,48,67]
**L^95^**	** 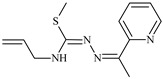 **	≥100	1.4	≥100	47.5	13.0	[46,47,48,73]
**L^96^**	** 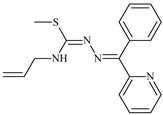 **	-	0.9	30.1	≥100	18.0	[46,47,48,67]
**L^97^**	** 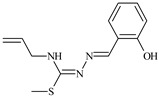 **	-	≥100	70.6	≥100	≥100	[46,47,48,67,73]
**L^97^·HI**			≥100	≥100	≥100	≥100	[46,47,48]
**L^98^**	** 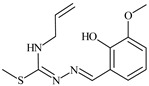 **	8.8	35.9	44.7	5.2	≥100	[46,47,48,67,73,82]
**L^98^·HI**		7.7	72.7	126.3	4.0	≥100	
**L^99^·HI**	** 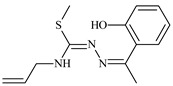 **	8.7	≥100	≥100	≥100	≥100	[46,57,67,82]
**L^100^**	** 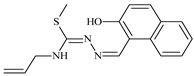 **	-	≥100	99.8	90	53	[46,67,82]
**L^101^**	** 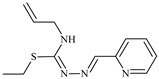 **	51.2	14.2	≥100	81.4	≥100	[46,67,82]
**L^102^**	** 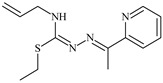 **	≥100	15.5	≥100	≥100	≥100	[46,67,73,82]
**L^102^·HI**		41.2	0.01	0.7	≥100	24	[46,57]
**L^103^·HI**	** 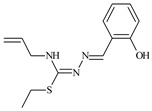 **	13.7	88.0	≥100	≥100	≥100	[46,67,82]
**L^104^**	** 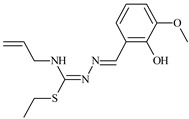 **	6.9	≥100	≥100	≥100	≥100	[46,57,67,73,82]
**L^104^·HI**		8.6	0.4	1.0	1.4	1.0	[46]
**L^105^·HI**	** 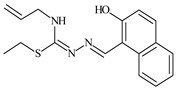 **	6.2	≥100	≥100	≥100	≥100	[46,57,67,82]
**L^106^**	** 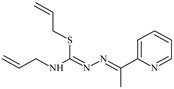 **	≥100	13.5	13.71	13.2	11.9	
**L^106^·HI**		49.9	0.47	1.18	1.45	1.5	[46]
**L^107^**	** 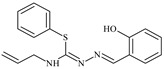 **	34.0	57.6	10.8	27.5	0.1	[47,48,67]
**L^108^**	** 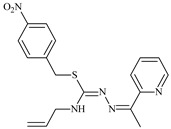 **	≥100	≥100	≥100	≥100	≥100	[46,57,67,74]
**L^108^·HBr**	** 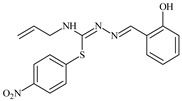 **	31.0	48.5	≥100	≥100	≥100	[46,67,74]
**L^109^**	** 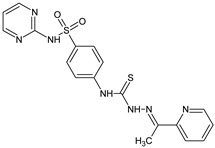 **	29.0	≥100	≥100	≥100	≥100	[47,48]

Average results of three experiments, SEM < ±3%.

**Table 4:** Antiproliferative activity of the coordination compounds against HeLa, RD, and BxPC-3 cancer cell lines, as well as MDCK cell lines. ABTS^•+^ scavenging activity of the 3*d* metal coordination compound.

	Formula	ABTS IC_50_ (µM)	BxPC-3 IC_50_ (µM)	RD IC_50_ (µM)	HeLa IC_50_ (µM)	MDCK IC_50_ (µM)	Reference
**5.1**	**Co(L^2^-H)_2_(CH_3_COO)**	≥100	≥100	≥100	≥100	≥100	[47,48]
**5.2**	**Ni(L^5^_)2_Cl_2_**	-	3.6	2.92	≥100	≥100	[46,47,48,83]
**5.3**	**Ni(L^5^-H)_2_**	-	4.9	11.81	≥100	≥100	
**5.4**	**Co(L^5^-H)_2_Cl**	-	4.1	≥100	14.2	≥100	
**5.5**	**Cu(L^5^_)_(NO_3_)_2_**	-	≤0.1	0.12	0.6	6	
**5.6**	**Cu(L^5^-H)Cl**	-	≤0.1	0.13	0.6	3	
**5.7**	**Cu(L^5^-H)Br**	-	≤0.1	0.2	0.3	3	
**5.8**	**Cu(L^5^-H)CH_3_COO**	-	≤0.1	0.05	0.2	2	
**5.9**	**Cu(Im)(L^5^-H)NO_3_**	-	0.1	0.2	4	3	
**5.10**	**Cu(3.5-Lut)(L^5^-H)NO_3_**	-	≤0.1	0.1	0.5	0.3	
**6.1**	**Cu(L^6^)(NO_3_)_2_**	29.8	0.18	0.09	2.2	3	[46,47,48,83]
**6.2**	**Cu(L^6^-H)(CH_3_COO)**	26.8	≤0.1	0.1	1.3	0.5	
**6.3**	**Cu(L^6^-H)Cl**	≥100	≤0.1	≤0.1	0.5	0.2	
**6.4**	**Cu(L^6^-H)Br**	≥100	≤0.1	≤0.1	0.4	0.4	
**6.5**	**(Cu(L^6^-H))_2_SO_4_**	38.7	≤0.1	≤0.1	0.4	0.1	
**6.6**	**Cu(4-Pic)(L^6^-H)(NO_3_)**	-	≤0.01	0.5	1.5	1.6	
**6.7**	**Cu(2.2′-Bpy)(L^6^-H)(NO_3_)**	-	≤0.01	1.29	1.9	1.4	
**6.8**	**Cu(1.10-Phen)(L^6^-H)(NO_3_)**	-	0.14	1.1	0.7	3.2	
**7.1**	**Ni(L^7^)_2_(NO_3_)_2_**	-	≥100	≥100	≥100	≥100	[46,47,48,83]
**7.2**	**Cu(L^7^-H)(NO_3_)**	≥100	0.15	0.3	1.4	1.2	
**7.3**	**Cu(L^7^-H)Cl**	52.9	≤0.1	0.1	0.02	1	
**12.1**	**Cu(L^12^-H)Cl**	51.8	0.6	0.5	1.4	2.5	[46,47,48,83]
**12.2**	**Cu(L^12^-H)Br**	52.7	0.3	0.4	1.4	1.7	
**12.3**	**Cu(L^12^-H)(NO_3_)**	76.2	≤0.1	0.5	1.3	6	
**12.4**	**Cu(L^12^-2H)H_2_O**	44.9	0.1	0.5	4.9	2.1	
**12.5**	**Co(L^12^-H)_2_Cl**	21.4	≥100	≥100	≥100	≥100	
**12.6**	**Ni(L^12^)(L^12^-H)Cl**	3.6	≥100	≥100	≥100	≥100	
**12.7**	**Zn(L^12^-H)Cl**	9.2	≥100	11.3	12.7	38.6	
**12.8**	**Fe(L^12^-H)_2_(NO_3_)**	2.4	≥100	79.7	37.5	24.1	
**12.9**	**Cu(Im)(L^12^-H)(NO_3_)**	5.8	0.02	1.8	7	12	
**12.10**	**Cu(3.5Br_2_Py)(L^12^-H)(NO_3_)** **where 3.5Br_2_Py is** ** 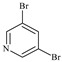 **	7.5	0.05	1.03	5	9	
**14.1**	**Cu(L^14^-H)Cl**	≥100	0.8	1.7	2	10	[46,47,48,81,83]
**15.1**	**Cu(L^15^-H)Cl**	68.3	≤0.1	0.1	0.2	1.3	[46,47,48,70,74,83]
**16.1**	**Cu(L^16^-H)NO_3_·H_2_O**	18.7	10.47	0.36	92	84	[46,47,48,70,74,81]
**16.2**	**Cu(Py)(L^16^-H)NO_3_**		≤0.1	0.1	1.48	1.5	
**16.3**	**Cu(L^16^-2H)H_2_O**	≥100	2.25	10.4	1.2	≥100	
**16.4**	**Cu(L^16^-H)Br**	≥100	1.2	12.2	3.7	39.6	
**16.5**	**Co(L^16^-H)_2_NO_3_**	59.4	96.5	≥100	28.79	≥100	
**17.1**	**Cu(L^17^-H)NO_3_**	7.1	0.1	0.6	1.5	1.5	[46,47,48,70,74,83]
**19.1**	**Cu(L^19^-H)NO_3_**	8.6	0.3	4.6	13	25	
**20.1**	**Cu(L^20^-H)Cl**	14.2	1.2	1.1	3.9	15.0	[46,47,48,74,81]
**20.2**	**Cu(L^20^-H)NO_3_**	14.4	≥100	6.8	8	9	
**20.3**	**Co(L^20^-H)_2_NO_3_**	8.5	≥100	23.3	65.3	≥100	
**20.4**	**Ni(L^20^)(L^20^-H)Cl**	8.5	≥100	36.1	90.0	≥100	
**20.5**	**Cr(L^20^-H)_2_NO_3_**	0.9	≥100	≥100	≥100	≥100	
**21.1**	**Cu(L^21^-H)Cl**	4.5	0.5	0.2	2.1	4	[47,48,49]
**21.2**	**Cu(Str)(L^21^-H)Cl** **where Str is** ** 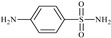 **	6.2	1.6	1.3	0.4	12	
**33.1**	**Cu(1.10-Phen)(L^33^-2H)**	11.8	2.0	10.9	4.6	13.0	[47,48,72]
**37.1**	**Cu(L^37^-H)(NO_3_)**	19.6	2.6	19.2	3.5	60.2	
**37.2**	**Cu(L^37^-H)Br**	38.3	9.3	11.2	59.7	15.1	
**37.3**	**Cu(L^37^-H)Cl**	27.2	6.2	9.3	10.9	12.5	
**37.4**	**Cu_2_(L^37^-H)_2_SO_4_**	13.2	16.97	≥100	76.7	≥100	
**37.5**	**Ni(L^37^-H)_2_**	7.2	≥100	≥100	≥100	≥100	
**37.6**	**Co(L^37^-H)_2_(NO_3_)**	14.2	≥100	≥100	≥100	≥100	
**44.1**	**Cu(L^44^-H)Br**	5.7	6.8	≥100	61.3	≥100	[47,48]
**44.2**	**Cu(L^44^-H)Cl**	15.7	16.5	≥100	≥100	≥100	
**44.3**	**Cu(L^44^-H)NO_3_·H_2_O**	9.5	1.6	≥100	5.6	≥100	
**44.4**	**Ni(L^44^-H)_2_**	2.9	≥100	≥100	≥100	≥100	
**44.5**	**Ni(L^44^-2H)(H_2_O)**	11.3	≥100	≥100	≥100	≥100	
**44.6**	**Co(L^44^-H)_2_Cl**	8.3	≥100	≥100	≥100	≥100	
**44.7**	**Co(L^44^-H)_2_(NO_3_)**	12.0	≥100	≥100	≥100	≥100	
**44.8**	**Fe(L^44^-H)_2_Cl**	5.4	≥100	≥100	≥100	≥100	
**44.9**	**Fe(L^44^-H)(NO_3_)_2_(H_2_O)**	18.6	55.6	≥100	73.2	≥100	
**44.10**	**Fe(L^44^-H)_2_(NO_3_)**	6.2	≥100	≥100	≥100	≥100	
**44.11**	**K_2_Mn(L^44^-2H)_2_**	10.1	23.8	≥100	6.2	≥100	
**44.12**	**Cu(L^44^-2H)(H_2_O)**	6.9	3.0	9.7	5.2	6.3	
**44.13**	**Cu(L^44^-H)ClO_4_**	8.1	24.7	≥100	24.5	≥100	
**49.1**	**Cu_2_(L^49^-H)_2_SO_4_**	≥100	2.5	12.7	8.6	11.4	
**49.2**	**Ni(L^49^-H)Cl**	9.6	≥100	≥100	≥100	≥100	
**54.1**	**Cu(L^54^-H)NO_3_·H_2_O**	15.7	4.2	≥100	3.5	≥100	[47,48]
**54.2**	**Ni(L^54^-H)Cl**	13.2	32.2	≥100	12.5	≥100	
**54.3**	**Cu(L^54^-H)Br**	9.3	6.8	2.0	2.8	9.0	
**54.4**	**Cu(L^54^-H)ClO_4_·4H_2_O**	30.8	11.8	9.3	4.7	13.0	
**54.5**	**Cu(L^54^-H)Cl**	7.5	6.6	≥100	21.5	≥100	
**54.6**	**Co(L^54^-H)_2_Cl**	7.5	≥100	≥100	≥100	≥100	
**54.7**	**Co(L^54^-H)_2_(NO_3_)**	11.1	≥100	≥100	≥100	≥100	
**54.8**	**Ni(L^54^-2H)(H_2_O)**	15.6	30.1	≥100	98.7	≥100	
**54.9**	**Cu(L^54^-2H)(H_2_O)**	8.5	13.1	≥100	30.3	≥100	
**57.1**	**Ni(L^57^-2H)(H_2_O)**	17.6	≥100	≥100	≥100	≥100	[47,48,49]
**57.2**	**Ni(L^57^-H)_2_**	12.7	≥100	≥100	≥100	≥100	
**60.1**	**Cu(L^60^-2H)(H_2_O)**	≥100	9.8	7.7	86.4	30.3	
**60.2**	**Cu(L^60^-H)Cl**	76.5	9.7	≥100	44.1	84.1	
**60.3**	**Cu(L^60^-H)Br**	-	4.81	≥100	28.6	-	
**60.4**	**Cu(L^60^-H)(NO_3_)**	91.9	5.9	6.7	77.8	12.5	
**60.5**	**Ni(L^60^-2H)(H_2_O)**	14.3	≥100	≥100	≥100	≥100	
**63.1**	**Cu(L^63^-H)NO_3_**	19.9	0.10	1.9	1.08	2.4	
**63.2**	**Cu(L^63^-H)Cl**	22.4	1.2	16.4	5.9	14.8	
**64.5**	**Cu(L^64^-H)Cl**	82.5	15.4	≥100	27.6	≥100	
**64.6**	**Cu(L^64^-H)NO_3_**	23.0	≤0.1	1.3	0.77	1.7	
**64.7**	**Co(L^64^-H)_2_Cl**	18.7	1.01	≥100	3.1	≥100	
**64.8**	**Ni(L^64^-H)Cl**	-	0.8	≥100	1.7	≥100	
**65.1**	**Cu(L^65^-H)NO_3_**	34.1	≥100	≥100	≥100	≥100	
**65.2**	**Co(L^65^-H)_2_Cl**	12.8	≥100	≥100	≥100	≥100	
**65.3**	**Fe(L^65^-H)_2_NO_3_**	10.4	≥100	≥100	≥100	≥100	
**66.1**	**Cu(L^66^-2H)(H_2_O)**	12.3	0.06	≥100	2.7	≥100	
**66.2**	**Cu(L^66^-H)Br**	15.9	9.2	≥100	28.0	≥100	
**66.3**	**(Cu(L^66^-H))SO_4_**	-	4.9	≥100	10.1	≥100	
**66.4**	**Ni(L^66^-H)_2_**	12.2	≥100	≥100	≥100	≥100	
**66.5**	**Fe(L^66^-H)_2_NO_3_**	20.4	8.1	≥100	19.8	≥100	
**66.6**	**Co(L^66^-H)_2_Cl**	9.7	≥100	≥100	≥100	≥100	
**67.1**	**Cu(L^67^-H)Cl**	27.5	1.0	7.7	8.5	11.3	
**67.2**	**Cu(L^67^-H)NO_3_**	18.8	6.4	15.5	16.6	21.2	
**69.1**	**Cu(L^69^-H)NO_3_**	14.6	≤0.1	0.9	0.3	2.2	
**69.2**	**Cu(L^69^-H)Cl**	20.2	0.1	1.1	5.0	2.5	
**70.1**	**Cu(L^70^-H)Cl**	≥100	3.9	13.1	12.4	28.8	
**70.2**	**Cu(L^70^-H)NO_3_**	24.7	0.07	0.2	0.5	0.3	
**70.3**	**Co(L^70^-H)_2_Cl**	3.7	≥100	≥100	≥100	≥100	
**72.1**	**Co(L^72^-H)_2_Cl**	9.0	≥100	≥100	≥100	≥100	
**73.1**	**Cu(L^73^-H)NO_3_**	25.3	6.1	≥100	27.3	≥100	
**73.2**	**Cu(L^73^-H)Cl**	40.5	9.4	19.7	26.1	26.6	
**74.1**	**Cu(L^74^-H)NO_3_**	12.0	0.11	1.2	1.10	2.1	
**74.2**	**Zn(L^74^-H)Cl**	-	6.0	≥100	21.0	≥100	
**74.3**	**Co(L^74^-H)_2_Cl**	50.3	11.5	14.0	≥100	≥100	
**75.1**	**Cu(L^75^-H)Cl**	17.6	0.1	2.6	1.2	1.5	
**76.1**	**Cu(L^76^-H)Cl**	19.9	≤0.1	1.4	1.06	2.96	
**76.2**	**(Cu(L^76^-H))_2_SO_4_**	-	1.6	1.6	8.2	11.6	
**76.3**	**Cu(L^76^-H)NO_3_**	20.1	0.1	20.0	1.1	≥100	
**76.4**	**Co(L^76^-H)_2_Cl**	≥100	9.4	≥100	8.5	≥100	
**78.1**	**Co(L^78^-H)_2_Cl**	14.8	≥100	≥100	≥100	≥100	
**78.2**	**(Cu(L^78^-H))_2_SO_4_**	-	2.0	≥100	5.6	≥100	
**79.1**	**Cu(L^79^-H)NO_3_**	54.6	4.8	≥100	10.4	≥100	
**79.2**	**Cu(L^79^-H)Cl**	38.5	3.11	14.9	11.6	13.9	
**80.1**	**Cu(L^80^-H)Cl**	6.6	1.1	0.3	6.5	0.1	[47,48,49]
**80.2**	**Cu(L^80^-H)Br**	9.1	0.5	0.3	1.0	0.1	
**80.3**	**Cu(L^80^-H)NO_3_·H_2_O**	8.0	0.4	0.5	0.9	0.0	
**80.4**	**Cu(L^80^-H)(CH3COO)·H_2_O·C_2_H_5_OH**	11.3	0.5	0.5	1.1	0.1	
**80.5**	**Cu(L^80^-H)(ClO_4_)·C_2_H_5_OH**	13.3	1.1	0.6	7.7	0.5	
**81.1**	**Cu(L^81^-H)NO_3_·H_2_O**	35.8	3.4	5.4	1.4	2.8	
**81.2**	**Cu(L^81^-H)CH_3_COO·H_2_O**	10.5	4.1	1.8	1.4	0.1	
**81.3**	**Cu(L^81^-H)ClO_4_·H_2_O**	11.5	4.3	2.4	2.5	0.1	
**81.4**	**Cu(L^81^-H)Br**	2.9	59.8	≥100	47.8	≥100	
**81.5**	**Cu(L^81^-H)Cl**	32.5	30.2	1.6	30.9	0.4	
**82.1**	**Co(L^82^-H)_2_NO_3_**	6.0	48.6	≥100	≥100	35.5	
**82.2**	**Co(L^82^-H)_2_Br**	6.1	51.1	≥100	≥100	≥100	
**82.3**	**Co(L^82^-H)_2_Cl**	6.2	≥100	≥100	≥100	≥100	
**82.4**	**Co(L^82^-H)_2_CH3COO**	7.7	14.7	≥100	≥100	≥100	
**82.5**	**Fe(L^82^-H)_2_Br**	8.8	≥100	≥100	≥100	≥100	
**83.1**	**Cu(L^83^-H)Cl**	24.3	≥100	≥100	≥100	≥100	
**83.2**	**Cu(L^83^-H)NO_3_**	23.3	≥100	≥100	≥100	≥100	
**83.3**	**Ni(L^83^-H)Cl**	24.0	≥100	≥100	≥100	≥100	
**83.4**	**Zn(L^83^-H)Cl**	-	≥100	≥100	≥100	≥100	
**84.1**	**Cu(L^84^-H)Br**	18.6	≥100	≥100	≥100	≥100	
**84.2**	**Cu(L^84^-H)Cl**	14.3	≥100	≥100	≥100	≥100	
**86.1**	**Co(L^86^-H)_2_Br·H_2_O**	17.1	≥100	≥100	≥100	≥100	
**87.1**	**Cu(L^87^-2H)(H_2_O)**	8.7	≥100	≥100	≥100	≥100	
**87.2**	**Cu(L^87^-H)(CH3COO)·H_2_O**	4.9	≥100	≥100	≥100	≥100	
**87.3**	**Cu(L^87^-H)(NO_3_)·H_2_O**	6.6	≥100	≥100	≥100	≥100	
**87.4**	**Cu(L^87^-H)(ClO_4_)·H_2_O**	2.4	≥100	≥100	≥100	≥100	
**87.5**	**Ni(L^87^-H)(NO_3_)·H_2_O**	14.5	≥100	≥100	≥100	≥100	
**87.6**	**Ni(L^87^-H)(CH3COO)**	13.2	≥100	≥100	≥100	≥100	
**87.7**	**Ni(L^87^-H)_2_·H_2_O·C_2_H_5_OH**	4.1	≥100	≥100	≥100	≥100	
**88.1**	**Cu(L^88^-H)Br·H_2_O**	27.2	≥100	≥100	≥100	≥100	
**88.2**	**Cu(L^88^-2H)(H_2_O)**	31.9	≥100	≥100	≥100	≥100	
**88.3**	**Cu(L^88^-H)ClO_4_·C_2_H_5_OH**	38.9	≥100	≥100	≥100	≥100	
**88.4**	**Cu(L^88^-2H)·H_2_O·C_2_H_5_OH**	15.0	≥100	≥100	≥100	≥100	
**88.5**	**Ni(L^88^-H)Cl**	28.8	≥100	≥100	≥100	≥100	
**88.6**	**Ni(L^88^-H)(CH3COO)·H_2_O**	22.3	≥100	≥100	≥100	≥100	
**88.7**	**Ni(L^88^-H)(NO_3_)·H_2_O**	12.3	≥100	≥100	≥100	≥100	
**89.1**	**Ni(L^89^-H)(CH3COO)·H_2_O**	6.9	≥100	≥100	≥100	≥100	
**90.1**	**Cu(L^90^-H)Cl**	17.1	≤0.1	0.03	0.4	1 ± 9	[47,48,84]
**90.2**	**Zn(L^90^-H)Cl**	6.7	62.0	42.8	0.4	10	
**94.1**	**Cu(L^94^)(NO_3_)_2_**	≥100	1.0	1.5	1.1	1.3	[46,47,48,67]
**94.2**	**Cu(L^94^)Cl_2_**	≥100	1.0	1.1	0.9	5	
**94.3**	**Cu(L^94^)Br_2_**	≥100	0.3	0.6	1.2	1.5	
**94.4**	**Cu(L^94^)(ClO_4_)_2_**	≥100	1.3	1.3	1.4	5.5	
**94.5**	**Cu(L^94^-H)CH_3_COO**	-	0.7	1.7	5.4	6.6	
**94.6**	**Zn(L^94^)_2_I_2_**	-	≥100	10.9	≥100	68	
**94.7**	**Ni(L^94^)_2_I_2_**	-	51.6	14.1	54	95	
**94.8**	**Ni(L^94^)_2_(ClO_4_)**	-	30.6	≥100	74.9	≥100	
**94.9**	**Co(L^94^-H)_2_I**	≥100	≥100	≥100	≥100	≥100	
**94.10**	**Co(L^94^-H)_2_Cl**	-	48.1	15.8	2.1	≥100	
**94.11**	**Co(L^94^-H)_2_(NO_3_)**	≥100	≥100	57.0	≥100	≥100	
**94.12**	**Cu(L^95^)SO_4_**	≥100	≤0.1	0.7	2	2	[46,47,48,73]
**95.2**	**Cu(L^95^)Cl_2_**	≥100	≤0.1	0.1	3	1	
**95.3**	**Cu(L^95^)Br_2_**	≥100	≤0.1	≤0.1	0.5	0.3	
**95.4**	**Ni(L^95^)_2_(ClO_4_)_2_**	25.1	2.5	6.4	10.1	7.3	
**95.5**	**Ni(L^95^)_2_(NO_3_)_2_**	21.5	16.4	≥100	25.5	≥100	
**95.6**	**Co(L^95^-H)_2_NO_3_**	≥100	12.9	7.5	12.8	18.5	
**95.7**	**Co(L^95^-H)_2_I**	≥100	21.8	11.2	10.9	18.8	
**95.8**	**Cu(L^95^)(NO_3_)_2_**	≥100	≤0.1	0.1	1.0	0.7	
**95.9**	**Fe(L^95^-H)_2_Br**	≥100	8.5	10.7	3.4	11.0	
**95.10**	**Co(L^95^-H)_2_Cl**	≥100	≥100	12.6	92	42	
**96.1**	**Cu(L^96^)Br_2_**	45.9	0.2	1.3	0.3	1.64	[46,47,48,67]
**96.2**	**Cu(L^96^-H)** **(CH3COO)**	≥100	2.8	2.5	1	3	
**96.3**	**Cu(L^96^)Cl_2_**	≥100	≥100	13.4	≥100	36.3	
**96.4**	**Ni(L^96^)_2_** **(NO_3_)_2_**	21.1	1.3	8.3	2.9	14.4	
**96.5**	**Ni(L^96^)_2_** **(ClO_4_)_2_**	22.0	3.0	9.3	4.3	10.0	
**96.6**	**Co(L^96^-H)_2_** **(CH3COO)**	≥100	7.3	1.4	1	12.4	
**96.7**	**Co(L^96^-H)_2_** **NO_3_**	≥100	1.3	1.1	0.8	1.9	
**96.8**	**Zn(L^96^)I_2_**	55.7	2.5	12.2	1.4	11.1	
**96.9**	**Fe(L^97^-H)_2_NO_3_**	10.0	≥100	0.9	15	75	[46,47,48]
**97.2**	**Co(L^97^-H)_2_NO_3_**	7.1	≥100	49.3	≥100	≥100	
**97.3**	**Co(L^97^-H)_2_Cl**	23.2	≥100	≥100	≥100	≥100	
**97.4**	**Co(L^97^-H)_2_I**	21.7	≥100	≥100	≥100	≥100	
**97.5**	**Cr(L^97^-H)_2_NO_3_**	1.2	≥100	≥100	≥100	≥100	
**97.6**	**Ni(L^97^)(L^97^-H)ClO_4_**	-	22.4	≥100	91.1	≥100	
**97.7**	**Cu(L^97^-H)CH_3_COO**	-	0.4	11.1	3.1	13.1	
**97.8**	**Cu(L^97^-H)Br**	-	0.6	5.2	1.6	12.7	
**97.9**	**Cu(L^97^-H)Cl**	-	0.5	1.2	6.2	13.56	
**97.10**	**Cu(Py)(L^97^-H)NO_3_**	46.6	1.28	11.4	5.0	12.2	
**97.11**	**Cu(3-Pic)(L^97^-H)NO_3_**	53.1	0.7	13.8	9.4	16.9	
**97.12**	**Cu(Im)(L^97^-H)NO_3_**	48.9	2.8	12.1	4.7	27.3	
**97.13**	**Cu(3.4-Lut)(L^97^-H)NO_3_**	47.0	6.3	10.2	71.2	12.3	
**97.14**	**Cu(1.10-Phen)(L^97^-H)NO_3_**	11.6	0.96	1.28	2.7	1.34	
**97.15**	**Cu(L^98^H)Cl**	20.9	0.4	1.2	1.0	1.3	[46,47,48,67,73,82]
**98.2**	**Cu(L^98^-H)NO_3_**	38.9	3.7	1.19	1.3	4	
**98.3**	**Ni(L^98^)(L^98^-H)NO_3_**	15.5	≥100	≥100	≥100	30	
**98.4**	**Ni(L^98^)(L^98^-H)I**	8.3	15.4	9.7	4.3	12	
**98.5**	**Co(L^98^-H)_2_NO_3_**	20.9	≥100	≥100	≥100	≥100	
**98.6**	**Co(L^98^-H)_2_I**	19.2	≥100	≥100	≥100	≥100	
**98.7**	**Co(L^98^-H)_2_Cl**	23.1	≥100	≥100	≥100	≥100	
**98.8**	**Fe(L^98^-H)_2_NO_3_**	9.6	81.0	1.1	≥100	≥100	
**98.9**	**Cu(L^99^-H)Cl**	13.1	0.9	10.9	2.3	11.8	[46,57,67,82]
**99.2**	**Cu(L^99^-H)NO_3_**	12.2	2.5	12.5	14.5	12.2	
**99.3**	**Cu(L^99^-H)ClO_4_**	16.3	1.2	3.2	6.6	13.3	
**99.4**	**Cu(L^99^-H)(CH3COO)**	19.8	0.8	1.3	1.7	4.9	
**99.5**	**Ni(L^99^-H)_2_**	6.0	78.3	≥100	≥100	≥100	
**99.6**	**Co(L^99^-H)_2_NO_3_**	14.5	8.5	≥100	23.8	≥100	
**99.7**	**Co(L^99^-H)_2_Br**	7.5	5.0	10.7	≥100	27.2	
**99.8**	**Co(L^99^-H)_2_I**	15.3	35.9	≥100	32.1	≥100	
**99.9**	**Fe(L^99^-H)_2_NO_3_**	2.1	34.1	≥100	28.3	≥100	
**100.1**	**Cu(L^100^-H)ClO_4_**	11.9	1.3	12.5	3.9	14.7	[46,67,82]
**100.2**	**Ni(L^100^)(L^100^-H)ClO_4_**	5.2	9.3	≥100	44.0	≥100	
**100.3**	**Ni(L^100^-H)_2_**	8.6	0.72	9.8	3.5	12.6	
**100.4**	**Co(L^100^-H)_2_NO_3_**	16.5	44.3	≥100	≥100	≥100	
**100.5**	**Co(L^100^-H)_2_I**	-	≥100	117.5	57.7	≥100	
**100.6**	**Cr(L^100^-H)_2_NO_3_**	1.4	≥100	≥100	≥100	≥100	
**101.1**	**Cu(L^101^-H)Cl**	108.0	≤0.1	≤0.1	1.5	0.4	[46,67,82]
**101.2**	**Co(L^101^-H)_2_I**	≥100	11.3	11.1	14.0	16.9	
**102.1**	**Co(L^102^-H)_2_I**	≥100	4.1	1.2	1.9	5.6	[46,67,73,82]
**102.2**	**Ni(L^102^)_2_I_2_**	16.7	5.2	≥100	6.4	≥100	
**102.3**	**Cu(L^102^)Cl_2_**	-	≤0.01	0.2	0.5	1.4	
**102.4**	**Cu(L^102^)(NO_3_)_2_**	-	0.04	0.14	0.3	1.3	
**103.1**	**Cu(L^103^-H)NO_3_**	27.9	1.0	5.5	13	1.6	[46,67,82]
**103.2**	**Cu(L^103^-H)Br**	38.6	21.8	11.8	37.1	33.4	
**103.3**	**Ni(L^103^)(L^103^-H)ClO_4_**	8.0	0.8	1.4	1.7	1.1	
**103.4**	**Fe(L^103^-H)_2_NO_3_**	12.5	1.3	≥100	2.2	≥100	
**103.5**	**Co(L^103^-H)_2_NO_3_**	19.3	≥100	≥100	≥100	≥100	
**103.6**	**Cu(L^103^-H)Cl**	13.7	≥100	12.6	14	12	
**103.7**	**Ni(L^103^)(L^103^-H)I**	6.2	69.0	56.7	82.6	≥100	
**103.8**	**Co(L^103^-H)_2_I**	30.1	19.1	36.5	≥100	28.6	
**104.1**	**Co(L^104^-H)_2_I**	19.0	≥100	≥100	≥100	≥100	[46,57,67,73,82]
**104.2**	**Ni(L^104^-H)_2_**	12.0	≥100	≥100	≥100	≥100	
**104.3**	**Co(L^104^-H)_2_Br**	17.9	≥100	≥100	≥100	≥100	
**104.4**	**Co(L^104^-H)_2_(CH3COO)**	23.2	≥100	≥100	≥100	≥100	
**104.5**	**Co(L^104^-H)_2_NO_3_**	21.7	≥100	≥100	≥100	≥100	
**104.6**	**Fe(L^104^-H)_2_NO_3_**	4.0	3.7	-	9.8	-	
**105.1**	**Cu(L^105^-H)Cl**	-	1.16	6.7	7.9	20.0	[46,57,67,82]
**105.2**	**Cu(L^105^-H)NO_3_**	-	0.9	18.8	9.3	12.7	
**105.3**	**Co(L^105^-H)_2_I**	16.0	≥100	≥100	≥100	≥100	
**106.1**	**Cu(L^106^)Br_2_**	65.6	≤0.01	1.3	0.39	1.23	[46]
**106.2**	**Cu(L^106^)(NO_3_)_2_**	25.1	0.040	0.14	0.3	1.3	
**106.3**	**Cu(L^106^)Cl_2_**	65.5	≤0.01	0.2	0.5	1.4	
**107.1**	**Cu(L^107^-H)Cl**	≥100	1.5	1.2	1.1	0.0	[47,48,67]
**107.2**	**Co(L^107^-H)_2_Cl**	≥100	7.1	≥100	12.5	≥100	
**108.1**	**Cu(L^108^)Br_2_**	≥100	8.5	1.4	16.6	2.39	[46,57,67,74]
**108.2**	**Ni(L^108^)_2_Br_2_**	88.7	32.2	≥100	≥100	≥100	
**108.3**	**Fe(L^108^-H)_2_NO_3_**	≥100	3.1	2.0	5.5	1.1	
**108.4**	**Co(L^108^-H)_2_Br**	-	10.7	12.4	22.9	24.6	
**109.1**	**Ni(L^109^-H)_2_**	48.8	88.8	≥100	16.7	≥100	[46,67,74]
**109.2**	**Co(L^109^-H)_2_NO_3_**	≥100	21.9	≥100	≥100	≥100	

## Data Availability

No new data were created or analyzed in this study. Data sharing is not applicable to this article.
